# Forming N-heterocyclic carbene monolayers: not all deposition methods are the same†

**DOI:** 10.1039/d4nr04428b

**Published:** 2025-01-24

**Authors:** Aruna Chandran, Nathaniel L. Dominique, Gurkiran Kaur, Vincent Clark, Phattananawee Nalaoh, Lilian Chinenye Ekowo, Isabel M. Jensen, Mark D. Aloisio, Cathleen M. Crudden, Netzahualcóyotl Arroyo-Currás, David M. Jenkins, Jon P. Camden

**Affiliations:** a Department of Chemistry and Biochemistry, University of Notre Dame Notre Dame IN 46556 USA jon.camden@nd.edu; b Department of Chemistry, University of Tennessee Knoxville TN 37996 USA jenkins@ion.chem.utk.edu; c Chemistry-Biology Interface Program, Zanvyl Krieger School of Arts & Sciences, Johns Hopkins University Baltimore MD 21218 USA; d Department of Chemistry, Queen's University 90 Bader Lane Kingston Ontario K7L 3N6 Canada; e Carbon to Metal Coating Institute, C2MCI, Queen's University 90 Bader Lane Kingston Ontario K7L 3N6 Canada; f Institute of Transformative Bio-Molecules (WPI-ITbM), Nagoya University Nagoya 464-8601 Japan; g Department of Pharmacology and Molecular Sciences, Johns Hopkins University School of Medicine Baltimore MD 21205 USA

## Abstract

N-Heterocyclic carbenes (NHCs) are unrivaled in their ability to form persistent and tunable monolayers on noble metal surfaces, with disciplines from heterogeneous catalysis to microelectronics fabrication rapidly adopting this technology. It is currently assumed that different NHC monolayer preparation protocols yield equivalent surfaces; however, a direct comparison of the leading synthetic protocols is yet to validate this assumption. Herein, we explore the binding of NHC ligands to gold (Au) surfaces prepared using the five most common NHC deposition methods and discover significant differences in the resulting monolayer composition and structure. In this work, NHC-Au systems are prepared according to literature procedures starting from either the free carbene, the CO_2_ adduct, the bicarbonate salt, or the triflate salt. The resulting surfaces are characterized with surface-enhanced Raman spectroscopy (SERS), laser desorption/ionization mass spectrometry (LDI-MS), electrochemistry, and X-ray photoelectron spectroscopy (XPS). These data indicate that the free carbene, vacuum annealing, and solvent annealing methods form chemisorbed NHC monolayers, as expected; however, the solution phase methods without annealing yield surfaces with a fundamentally different character. Although XPS is widely used to confirm the binding of NHCs to metal surfaces, it does not capture the differences in these deposition procedures and should be used with caution. Taken together, these results reveal a significant variation of the NHC surface structure as a function of deposition procedure and provide a critical benchmark to govern the design and preparation of future NHC monolayer systems.

## Abstract 2

The graphic illustrates that the binding of N-heterocyclic carbenes to gold surfaces is influenced by both temperature and choice of precursor.

## Graphical abstract

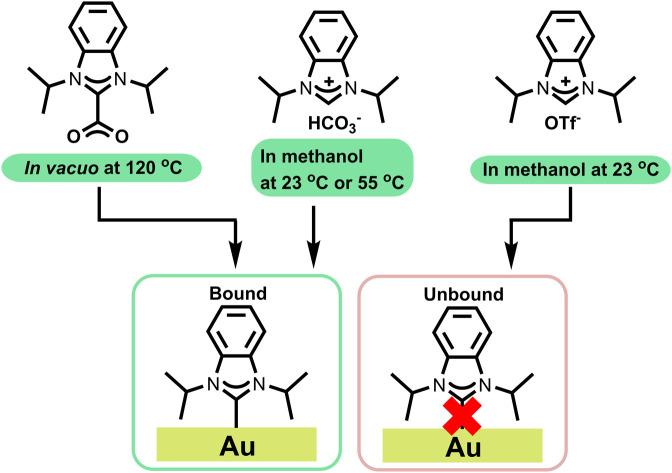

## Introduction

N-Heterocyclic carbene (NHC) ligands recently emerged from the field of homogeneous organometallic chemistry as a disruptive technology for the functionalization of noble metal surfaces.^1–5^ The unparalleled binding and tunability of NHCs enables recent applications in the electrocatalytic reduction of CO_2_,^6–10^ electronic devices,^11,12^ and micropatterning for electronics fabrication,^13–17^ to name a few examples. While NHCs provide a promising passivation platform, established standards or benchmarks for NHC monolayer quality and performance are lacking. Furthermore, the number of NHC monolayer synthesis protocols is proliferating and the resulting NHC-Au systems are generally considered equivalent; however, the work presented here reveals significant differences even between the standard methods. Considering the rapid expansion of NHC functionalized surfaces, new benchmarks are needed to evaluate both established and emerging methods.

The free carbene method, originally established by Fairlamb and Chechik,^18^ is the oldest and most widely used method to bind NHCs to metal surfaces.^6,18–37^ In this inert-atmosphere method, a free carbene is generated using a non-nucleophilic strong base to deprotonate the benzimidazolium or imidazolium precursor.^18–20^ The free carbene is then exposed to a gold surface allowing the NHC monolayer to form spontaneously. NHC monolayers created *via* this method are well characterized^20^ and are widely used for heterogeneous catalysis applications.^6,19,21,22,24,38^

Since the discovery of the free carbene method, complementary chemistries have been developed to form NHC monolayers that side-step the requirements of air-free synthesis during monolayer formation. The most common alternative methods involve heating a masked NHC precursor in the form of CO_2_ adducts^16,39–55^ or bicarbonate salts^14,16,26,41,43,48,56–62^ under vacuum to form high-quality NHC monolayers. Monolayers prepared *via* these methods have been characterized extensively,^40,50,55,58,62^ and are attracting interest for microcontact printing^14,16^ and surface-enhanced Raman spectroscopy (SERS)^42,43^ applications.

Both the free carbene and vacuum-based CO_2_/bicarbonate salt methods require specialized equipment, which motivated the development of benchtop protocols to form NHC monolayers on gold substrates. To this end, NHC bicarbonate salts^15,35,62–66^ and triflate/mesylate salts^67–70^ dissolved in methanol have been incubated with Au surfaces to spontaneously form monolayers under ambient conditions. Electrochemical methods to form monolayers have also been employed with related NHC precursors.^29^ Monolayers prepared *via* these benchtop procedures are widely used in electrochemistry,^67,68,70^ surface plasmon resonance sensors,^63,64^ and surface-grafted polymers.^69^ When taken together, the proliferation of methods to prepare NHC monolayers for applied research motivates the systematic study of deposition methods undertaken here.

**Fig. 1:**
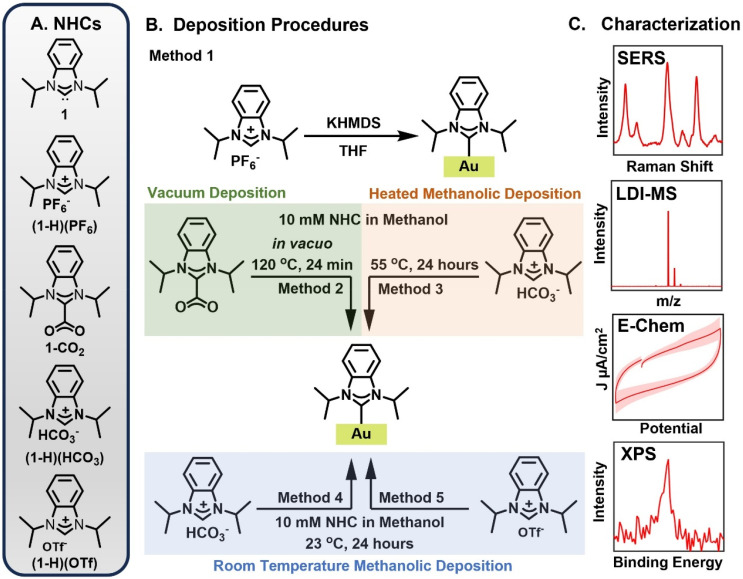
Schematic overview of experimental procedures. (A) NHC precursors derived from ligand 1. (B) Deposition procedures explored in this work. In the free carbene deposition, (1-H)(PF_6_) is deprotonated *via* potassium bis(trimethylsilyl)amide (KHMDS) and the resulting free carbene is exposed to a gold mirror under inert atmosphere (Method 1). In the vacuum deposition method, 1-CO_2_ is heated under vacuum atop a gold mirror (Method 2). In the methanolic deposition at 55 °C, the gold mirror is heated in a methanolic solution of (1-H)(HCO_3_) for 24 hours (Method 3). For the methanolic depositions at room temperature, (1-H)(HCO_3_) (Method 4) and (1-H)(OTf) (Method 5) are dissolved in methanol and exposed to a gold surface for 24 hours. (C) The resulting NHC based overlayers were characterized using surface-enhanced Raman spectroscopy (SERS), laser desorption/ionization mass spectrometry (LDI-MS), electrochemical methods (E-Chem), and X-ray photoelectron spectroscopy (XPS). A comprehensive evaluation of additional precursor/deposition protocol combinations is provided in the ESI.†

In this manuscript, we present a systematic study of NHC binding to gold surfaces as a function of the monolayer synthesis protocol. The archetypal NHC architecture, a benzimidazolylidene with isopropyl groups,^3^ was used as a model system to form NHC monolayers *via* the free carbene, vacuum annealing, and benchtop solution phase methods (Fig. 1). We characterized the resulting NHC based overlayers using a suite of surface-sensitive characterization methods. While NHC overlayers formed *via* these different methods are often assumed to be equivalent, our results illustrate dramatic differences in the overlayer as a function of the synthetic protocol. Only the NHC-Au overlayers formed *via* the free carbene, vacuum annealing, and solution phase annealing methods can be unambiguously attributed to a chemisorbed carbene. These results show that the choice of monolayer synthesis protocol will play an essential role in emerging NHC applications that rely on this chemistry.

## Results and discussion

NHC-Au overlayers were prepared according to established procedures (Fig. 1A and B) for the free carbene deposition of (1-H)(PF_6_) (Method 1),^19,30^ the vacuum deposition of 1-CO_2_ at 120 °C (Method 2),^42,43^ the heated solution phase benchtop deposition of (1-H)(HCO_3_) (Method 3),^71^ and the room temperature solution phase benchtop deposition of (1-H)(HCO_3_) (Method 4)^62^ and (1-H)(OTf) (Method 5).^68^ Experimental results for additional combinations of precursor/deposition protocols are provided in the ESI.†

### SERS characterization of NHC-Au overlayers

**Fig. 2:**
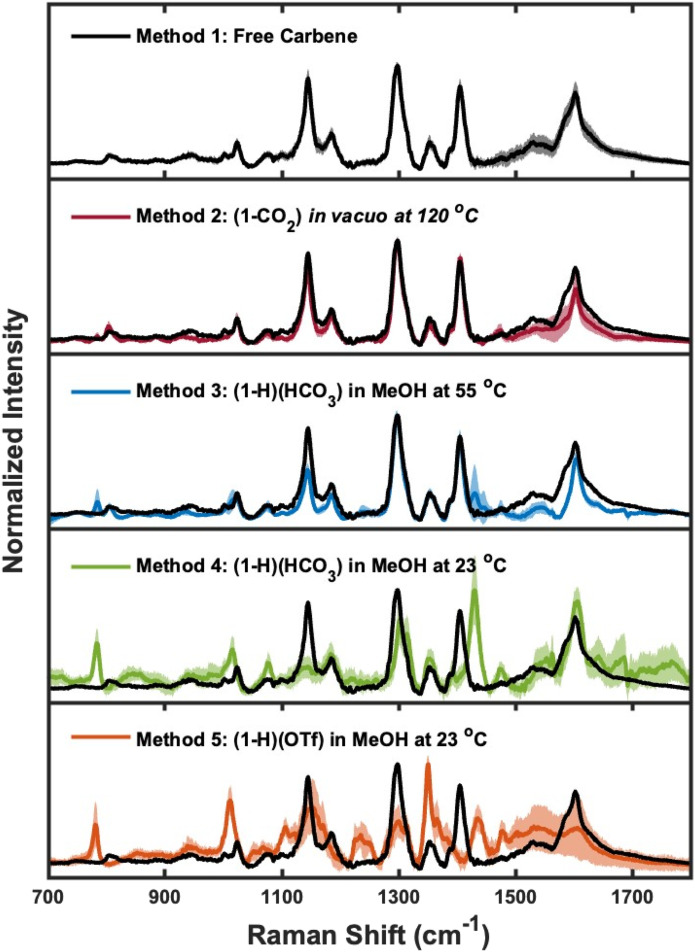
SER spectra of NHC-Au overlayers. SER spectra of the gold-NHC substrates after deposition *via* Method 1 (free carbene – black), Method 2 (1-CO_2_*in vacuo* at 120 °C – red), Method 3 ((1-H)(HCO_3_) in MeOH at 55 °C – blue), Method 4 ((1-H)(HCO_3_) in MeOH at 23 °C – green), and Method 5 ((1-H)(OTf) in MeOH at 23 °C – orange). The solid lines represent an average of 18 spectra (six measurements each from three replicate substrates) and the shaded regions represent the standard deviation of the replicates. The free carbene method (Method 1) is used as a benchmark and is overlayed on the other spectra. Methods 1, 2, and 3 all yield spectra in quantitative agreement within uncertainty. For Method 4 and Method 5, the characteristic bands at approximately 800 cm^−1^, 1300 cm^−1^, and 1400 cm^−1^ of the benchmark are absent, new spectral features appear, and large variation is observed. The spectra for Methods 4 and 5 also show decreased reproducibility, as evidenced by an increased standard deviation. Overall, these results indicate that the deposition method influences the resulting surface structure of NHC monolayers.

To study the binding of NHC ligands to gold surfaces, we characterized each NHC-Au overlayer with SERS. SERS is widely used to characterize ligand-gold systems and is capable of measuring chemical changes and ligand orientation.^72–76^ Monolayer formation *via* Method 1 (the free carbene method)^20^ and Method 2 (1-CO_2_*in vacuo* at 120 °C)^40,50,55,58^ has been extensively studied using numerous surface sensitive techniques (*e.g.* scanning tunneling microscopy (STM), HREELS), and our research groups have previously established the SERS signatures for monolayers formed *via* these methods.^30,42,43^ Therefore, we utilize the SERS data from Method 1 and Method 2 as benchmarks to compare with Methods 3–5. Fig. 2 displays the SER spectra obtained for each NHC-Au system. The spectrum for Method 3 is in excellent agreement with the spectra from Methods 1 and 2. Method 4 and Method 5 are dramatically different from benchmark SERS data as evidenced by the disappearance of NHC-Au signatures^43,77,78^ at approximately 800 cm^−1^, 1300 cm^−1^, and 1400 cm^−1^ and the appearance of new spectroscopic features at approximately 780 cm^−1^, 1011 cm^−1^, and 1430 cm^−1^.

SERS is a highly surface-specific technique and any differences in ligand binding can give rise to dramatic changes in SERS features.^75^ Method 2 (1-CO_2_*in vacuo* at 120 °C) and Method 3 ((1-H)(HCO_3_) in MeOH at 55 °C) produce SERS features that are in good agreement with Method 1 (free carbene) and past works from our groups.^42,43^ For Method 3, we observed the appearance of a new spectroscopic feature at ∼780 cm^−1^. Previous work from our groups combining measurements of NHC isotopologues and computational modeling illustrates that spectroscopic signatures in this region are diagnostic of NHC chemisorption or physisorption.^77,78^ The peak at ∼800 cm^−1^ arises from a NHC-Au vibrational mode with significant contribution from gold–carbon bond vibrations, whereas the new spectroscopic feature at ∼780 cm^−1^ is not present in published spectra of a chemisorbed NHC^43,77,78^ and suggests additional subpopulations of NHCs on the surface; however we caution that detailed spectroscopic studies with isotopologues and computational modeling would be required to fully assign this new vibrational mode. Dramatic differences are observed when comparing Method 4 ((1-H)(HCO_3_) in MeOH at 23 °C) and Method 5 ((1-H)(OTf) in MeOH at 23 °C) to the benchmark, with the most prominent bands from the benchmark spectrum absent. These SERS data illustrate that the ligand binding is heavily influenced by the deposition protocol and different surface structures form as the result of Method 4 and 5. The differences observed in the SER spectra from each method may arise due to either changes in NHC ligand orientation and/or binding,^42,43,75,77,78^ but orthogonal techniques will be needed to explore these differences fully.

### LDI-MS of NHC-Au overlayers

**Fig. 3:**
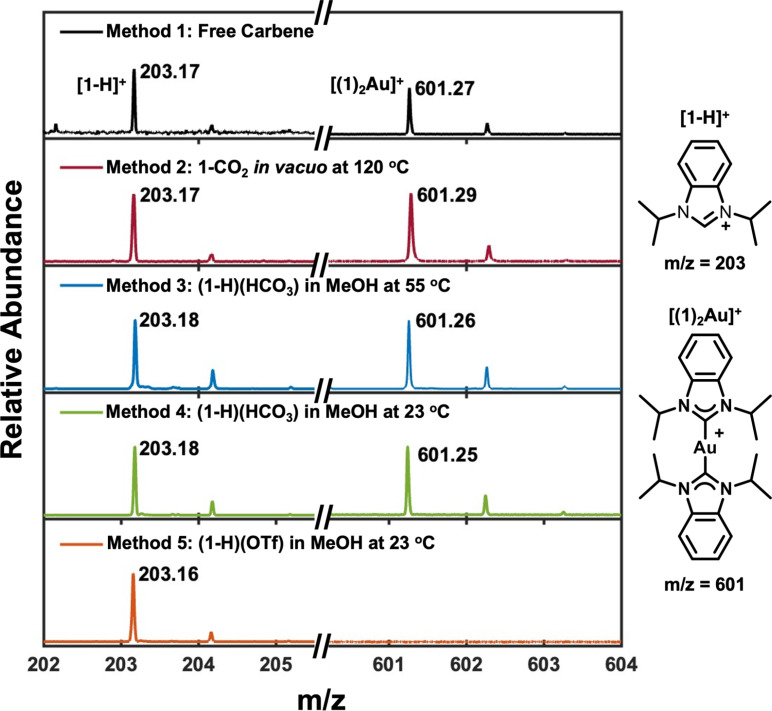
LDI-MS of NHC-Au overlayers. All NHC-Au systems were characterized using LDI-MS with NHC ion channels corresponding to [1-H]^+^ (left) and [(1)_2_Au]^+^ (right) displayed here. For all deposition methods, [1-H]^+^ ions are observed, indicating the presence of NHCs on the Au surface. Bis-NHC ions [(1)_2_Au]^+^ are observed for Method 1 (free carbene), Method 2 (1-CO_2_*in vacuo* at 120 °C), Method 3 ((1-H)(HCO_3_) in MeOH at 55 °C), and Method 4 ((1-H)(HCO_3_) in MeOH at 23 °C) and previous work shows these ions to be diagnostic of a chemisorbed carbene. [(1)_2_Au]^+^ ions are not detected for Method 5 ((1-H)(OTf) in MeOH at 23 °C). All spectra are normalized to [1-H]^+^ for 202–205 *m*/*z* region and to the Au_3_^+^ cluster at 590 *m*/*z* for the 601–604 *m*/*z* region.

LDI-mass spectra were collected for each NHC-Au system to measure NHC binding to Au (Fig. 3). This technique is widely used as a probe of ligands adsorbed to gold surfaces^79–81^ and was recently extended to the characterization of NHC monolayers.^54,77,82–84^ Previous reports illustrate that [(1)_2_Au]^+^ ions form as the result of nearest neighbor NHCs desorbing together from the monolayer^82^ and that these ions do not arise due to the LDI-MS plume chemistry;^54,82^ therefore their presence is indicative of chemisorbed NHCs. Fig. 3 illustrates that [1-H]^+^ ions were observed for all methods, indicating the presence of some form of NHC on the surface. However, [(1)_2_Au]^+^ ions, which indicate chemisorption, are only detected for Method 1 (free carbene), Method 2 (1-CO_2_*in vacuo* at 120 °C), Method 3 ((1-H)(HCO_3_) in MeOH at 55 °C), and Method 4 ((1-H)(HCO_3_) in MeOH at 23 °C). Similar to the SERS data, the presence of [(1)_2_Au]^+^ ions in Method 2 and 3 provides corroborating evidence that monolayers prepared *via* these methods share some of the same characteristics as monolayers prepared *via* the free carbene method. Surprisingly, [(1)_2_Au]^+^ ions were also observed for Method 4. The dramatic differences between SER spectra for Method 1 and Method 4 illustrates the importance of employing more than one technique for analysis of these overlayers and suggests that there are likely several different sub-populations of NHCs that form *via* Method 4. These populations may include physisorbed and chemisorbed NHCs, or different orientations, but the LDI-MS data suggest the presence of a reasonable quantity of chemisorbed NHC in overlayers formed from Method 4. Although [(1)_2_Au]^+^ ions were not observed for Method 5 ((1-H)(OTf) in MeOH at 23 °C), ions characteristic of a chemisorbed NHC were observed upon deposition of (1-H)(OTf) *in vacuo* at 120 °C (Fig. S4†). Therefore, the (1-H)(OTf) precursor can form chemisorbed NHCs with optimized conditions.

### Electrochemical characterization of NHC-Au overlayers

While SERS and LDI-MS measurements provide a qualitative measure of NHC ligand binding and orientation on the surface, complementary measurements are needed to assess the surface coverage. Monolayers on gold electrodes create a dielectric layer between the gold surface and the electrolyte, which may be modeled using a parallel plate capacitor.^85^ Previous research with this model has shown that a high degree of electrode passivation leads to low capacitive currents;^70,86^ therefore, we use these measurements to interrogate the degree of surface passivation.

**Fig. 4:**
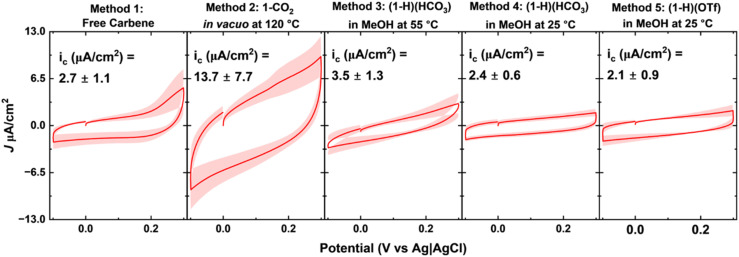
Measurement of capacitive currents following NHC-Au overlayer formation. To determine the degree of surface passivation, the capacitive currents of all NHC functionalized gold surfaces were measured. These results illustrate that Method 3 ((1-H)(HCO_3_) in MeOH at 55 °C), Method 4 ((1-H)(HCO_3_) in MeOH at 25 °C), and Method 5 ((1-H)(OTf) in MeOH at 25 °C) all passivate the electrode to a similar degree as Method 1 (free carbene) with low capacitive currents. Method 2 (1-CO_2_*in vacuo* at 120 °C), on the other hand, is characterized by relatively high capacitive currents, indicating a lower degree of surface passivation. As surface passivation is an indirect measurement of surface coverage, these measurements cannot distinguish between multiple subpopulations passivating the electrode surface. The solid lines and shaded regions represent the average and standard deviation of at least 4 samples, respectively. All capacitive currents were sampled at 0.1 V, using a voltage scanning rate of 100 mV s^−1^.

Capacitive current density measurements (Fig. 4) for NHC-Au electrodes reveal a low capacitive current of 2.7 μA cm^−2^ for the benchmark Method 1 (free carbene). Method 3 ((1-H)(HCO_3_) in MeOH at 55 °C), Method 4 ((1-H)(HCO_3_) in MeOH at 25 °C), and Method 5 ((1-H)(OTf) in MeOH at 25 °C) also produce low capacitive currents comparable to Method 1. Method 2 (1-CO_2_*in vacuo* at 120 °C), on the other hand, produces higher capacitive currents than the four other methods, suggesting incomplete surface passivation. While SERS and LDI-MS data for Method 1, Method 2, and Method 3 suggest that the NHC overlayers prepared by these methods are comparable, electrochemistry measurements illustrate that Method 1 and Method 3 passivate the gold surface more effectively than Method 2. However, treating gold surfaces with 1-CO_2_ under the same conditions as Method 3 (*i.e.* in MeOH at 55 °C), comparable capacitive currents are observed (Fig. S6 and S7†). Although Methods 4 and 5 produce low capacitive currents, SERS and LDI-MS data suggest that there are notable differences in these monolayers compared to Method 1, free carbene.

### XPS of NHC-Au overlayers

**Fig. 5:**
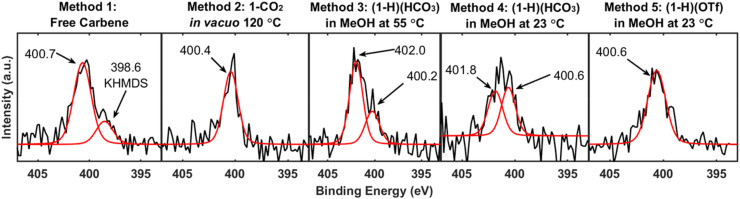
XPS of NHC-Au overlayers. XPS analysis of the N 1s region for NHC-based overlayers on gold substrates prepared using Methods 1–5. The N 1s peak for Method 1 (free carbene) is in excellent agreement with previous reports of chemisorbed NHCs on gold typically between 399.9 eV and 401.0 eV. The peak at 398.6 eV is attributed to residual KHMDS. While the N 1s peak positions for Method 2 (1-CO_2_*in vacuo* at 120 °C), and Method 5 ((1-H)(OTf) in MeOH at 23 °C) fall within the range for a chemisorbed NHC, one additional peak appears for Method 3 ((1-H)(HCO_3_) in MeOH at 55 °C) and Method 4 ((1-H)(HCO_3_) in MeOH at 23 °C).

X-ray photoelectron spectroscopy^87^ (XPS) is a prolific technique for the characterization of NHC-based overlayers on gold and is widely used to confirm chemisorption *via* observation of the N 1s peak.^59^ Prior research reports N 1s binding energies as occurring between 399.9 and 401.0 eV^41,46,48,53,58,69,70^ for chemisorbed NHCs. For completeness, we analyzed the prepared NHC-based overlayers by XPS, including survey scans and high-resolution element scans of the N 1s region of both NHC functionalized substrates and parallel control samples (Fig. S8–S17†). Fig. 5 displays the N 1s binding energy region for each method. Method 1, Method 2, and Method 5 all display N 1s peaks at positions customarily assigned to an NHC ligand bound to gold. Interestingly, SERS and LDI-MS reveal stark differences between Method 5 and the benchmark methods, but these differences are not observed in the XP spectra. In addition to the expected signal at 400.7 eV, the free carbene method displays a small shoulder on the main peak at 398.6 eV, which is attributed to residual KHMDS on the surface (Fig. S11†). While the residual KHMDS convolutes the XP spectrum for Method 1, KHMDS does not influence the SERS or LDI-MS data (Fig. S1 and S2†). The XP spectra for Method 3 ((1-H)(HCO_3_) in MeOH at 55 °C) and Method 4 ((1-H)(HCO_3_) in MeOH at 23 °C) both display one N 1s signal in agreement with an NHC bound to gold and an additional signal at >401 eV. Although these features are generally attributed to physisorbed NHC on the surface,^23,48,71^ ongoing XPS studies in our groups suggests that this peak assignment may need to be revisited. XPS results were unable to resolve the dramatic differences in NHC binding to Au observed *via* other methods (*i.e.* SERS and LDI-MS) illustrating the limitations of XPS when characterizing NHC systems. However, these studies point to the possibility of more than one species on the surface in some cases, including contamination by residual protonated base in the case of deposition of the free NHC (Method 1). While the effect of residual KHMDS on the NHC overlayer quality is the subject of active research, residual base on the gold surface will likely alter the overlayer packing density.

In a recent study by Ohto, Jae Yoon, and co-workers,^36^ a comparison of the free carbene, electrochemical, and a solution phase deposition at room temperature was conducted. Our study is in good agreement with their finding that the free carbene method produces higher quality NHC overlayers than solution phase methods at room temperature.

## Conclusions

In conclusion, the systematic investigation presented here reveals significant differences in the structure and quality of NHC-based overlayers on gold as a function of the synthesis protocol. For both bicarbonate salts and CO_2_ adducts, annealing the gold surface in methanol produces a chemisorbed NHC with a high degree of surface passivation as evidenced by SERS, LDI-MS, and capacitive current measurements. The benchtop solution-phase deposition methods at room temperature contrast sharply with solution and vacuum annealing samples. No evidence of a gold-bound NHC was detected in LDI-MS for the solution phase triflate salt depositions, however these surfaces were shown to be highly passivated by electrochemical measurements. While the bicarbonate salt solution phase deposition under ambient conditions produces a population of chemisorbed carbenes, qualitative differences in the SERS data illustrate the formation of sub-populations of different NHC surface structures. In general, thermal annealing during the deposition procedure and solution phase deposition were shown to be essential ingredients to form chemisorbed NHCs with a high degree of surface passivation. Lastly, although XPS is often considered to be diagnostic for NHC chemisorption, this technique is not always suitable for determining differences in NHC surface structure. XPS does, however, provide good insight into the presence of contaminant species in NHC on gold overlayers. Thus, it is clear that orthogonal methods are critical when analyzing NHC-based overlayers on gold. Follow-up studies deploying advanced methods like scanning tunneling microscopy will prove invaluable to acquire a comprehensive understanding of how each deposition procedure influences the surface structure. The results presented herein provide a benchmark for future NHC binding studies and illustrate that NHC deposition protocols are a critical variable when designing functional NHC monolayer systems.

## Summary of deposition protocols

To supplement the brief introduction of deposition protocols used to form NHC monolayers, we include a more detailed description of the deposition protocols here.

### General considerations

Synthetic procedures for NHC precursor preparation should be carefully chosen based on what surface contaminants are acceptable for a given application since trace contaminants from synthetic precursors, *e.g.* iodide^88^ and silver,^69^ accumulate onto metal surfaces. Likewise, the use of bromide^29^ or iodide^69^ containing precursors in the synthesis of benzimidazolium or imidazolium salts has been shown to deposit residual halides onto the metal surface. Attempts to remove iodide from NHC precursors using silver leaves enough residual silver contamination on the surface to detect *via* XPS.^69^ Therefore, careful planning of the NHC synthesis is essential to avoid surface contamination from common species like bromide, iodide, and metal ions.

### The free carbene method

Formation of NHC monolayers *via* the free carbene method involves the use of a non-nucleophilic strong base to deprotonate an NHC ligand precursor, *e.g.* a benzimidazolium salt, to form the free carbene. Because the free carbene is highly reactive, it is not stable under ambient conditions and these reactions must take place under an inert atmosphere. Typically, an imidazolium or benzimidazolium salt is reacted with potassium *tert*-butoxide^20^ or KHMDS^19^ in a dry solvent within a glovebox or using standard Schlenk chemistry techniques to maintain an inert atmosphere. Metal surfaces may then be exposed to the free carbene solution, and the NHC will bind spontaneously to metal atoms on the surface. This procedure is very general but requires specialized equipment and laborious experimental conditions. In addition, it is important to ensure that the residue from the base employed to generate the NHC does not contaminate the surface. For example, the use of potassium *tert*-butoxide to form free carbenes has been shown to leave residual potassium on the metal surface.^16,31^ As shown here and in other publications,^19^ residual amine from the base can also contaminate the surface.

### Vacuum based methods

To form an NHC monolayer using a vacuum based method, a masked carbene consisting of a CO_2_ adduct or bicarbonate salt is heated *in vacuo* to liberate CO_2_ or H_2_O and CO_2_, respectively. After loss of CO_2_ and H_2_O, a free carbene forms and binds with the metal surface. Masked carbenes are widely used in NHC chemistry and are stable under ambient conditions. For example, masked NHCs have been used in homogeneous organometallic chemistry to form NHC metal complexes using NHC bicarbonate salts and CO_2_ adducts.^89,90^

Initial approaches to form NHC monolayers using masked carbenes involved heating NHC-CO_2_ adducts in a dry solvent to form the free carbene, which was then exposed to a gold surface.^19^ Alternative vapor phase approaches were subsequently developed where the bicarbonate salt^62^ or CO_2_ adduct^40^ were sublimed in an ultrahigh vacuum chamber at mild temperatures. The free carbene forms within the vacuum chamber through this physical vapor deposition process and reacts with a metal surface placed in the line of sight of the NHC sublimation source. Because the main side products of this procedure are H_2_O and CO_2_, this process is known to produce exceptionally clean NHC monolayers and is highly favored for scanning tunneling microscopy (STM) studies.^40,41,45,46,49,53,55,58,60,62^ NHC monolayers have been formed on numerous metal substrates using this approach, including gold,^40,44,46,47,55,58,62^ silver,^46,53^ and copper.^46,53,60^

NHC monolayers may also be formed under rough vacuum conditions using masked NHCs. For example, our research groups recently used NHC-CO_2_ adducts heated under rough vacuum conditions to form NHC monolayers on nanostructured gold substrates for SERS and LDI-MS studies.^30,42,43,51,91^ Additionally, Glorius, Ravoo, and co-workers^16^ also demonstrated that masked NHCs deposited onto a gold surface *via* microcontact printing may be heated under vacuum to generate NHC functionalized films.

### Solution phase benchtop methods

Another approach to form NHC monolayers involves treating a metal surface with NHC bicarbonate salts,^62^ triflate salts,^67^ or mesylate salts^69^ in a suitable solvent (typically methanol^62^ or THF^88^). Bicarbonate salts are the most common precursor for this deposition procedure and are widely used for both fundamental and applied research studies.^15,56,62–64,66,92^ In a typical procedure, the bicarbonate salt is dissolved in methanol and exposed to a metal surface under ambient conditions for an extended time either at room temperature or at elevated temperatures.^62,93^ Recently, Braunschweig and Glorius^71^ reported a modified version of this procedure that involves heating a solution of NHC bicarbonate salt in ethanol in the presence of a gold surface. Triflate^67,68,70,94^ and mesylate salt^69^ procedures involve exposing a metal surface to a methanolic solution of the salt at room temperature. Solution based NHC procedures are especially popular for the preparation of NHC based biosensors.^67,68,94^

## Experimental methods

### Materials

NaOH pellets and HPLC grade methanol (>99.9%) were purchased from Sigma Aldrich (St Louis, MO, USA). Concentrated H_2_SO_4_, 30% H_2_O_2_, and reagent alcohol (89.5–91.5% ethanol, 4.0–5.0% methanol, and 4.5–5.5% isopropanol) were obtained from VWR (Radnor, PA, USA). Ag|AgCl (3 M KCl) reference electrodes and platinum wire counter electrodes were obtained from BASI (West Lafayette, IN, USA). Ultrapure water (18 MΩ) was prepared in house using a Barnstead system from Thermo Fisher (Waltham, Massachusetts, US). Tantalum clip electrodes were acquired from RedoxMe (Norrköping, Sweden).

### Au mirror preparation

Gold (Au) mirrors were prepared with 100 nm of gold and 5 nm of chromium deposited onto piranha acid-etched glass slides using a physical vapor deposition system equipped with a quartz crystal microbalance (Nano36, Kurt J. Lesker, Jefferson Hills, PA, USA). To clean the glass microscope slides piranha acid (caution, extremely hazardous) was used. Piranha acid was prepared *in situ* using concentrated H_2_SO_4_ and 30% H_2_O_2_ in a 4 : 1 ratio and used to clean the glass surface by immersion for 60 minutes. Piranha solution should never be stored, but instead properly disposed of after use. After 60 minutes, the cleaned mirrors were rinsed with water and reagent alcohol and dried under nitrogen. Then, 5 nm of chromium and 100 nm of gold were deposited *via* thermal evaporation with a base pressure of ∼10^−6^ torr. The Au mirrors were rinsed with reagent alcohol and water. Next, the mirrors were electrochemically cleaned in 0.5 M NaOH *via* 200 cycles of cyclic voltammetry from −0.3 to −1.6 V *vs.* Ag|AgCl (3 M KCl). Then the mirror was cycled in 0.5 M H_2_SO_4_ from 0 to 1.55 V for 25 cycles.^70^ The mirror was then washed with water and HPLC-grade methanol. The electrochemical cleaning process of the Au mirror was carried out utilizing either a CH Instruments model 600E Series Electrochemical Analyzer/Workstation with a multiplexer module or an Autolab PGSTAT204 with scan rates of 0.5 V s^−1^ or 1.25 V s^−1^, respectively.

### Free carbene deposition method

NHC monolayers were prepared *via* the free carbene method in a similar manner as reported in literature.^30^ A solution of free carbene was made by adding 1,3-diisopropyl benzimidazolium hexafluorophosphate, (1-H)(PF_6_), (0.100 g, 0.287 mmol, 1 eq.) to a 20 mL scintillation vial with 10 mL of THF followed by KHMDS (0.057 g, 0.287 mmol, 1eq.) in a glove box. The solution was stirred for 30 minutes prior to filtration through a Celite pad. Two drops of this free carbene solution in THF were then deposited onto an Au mirror. The vial was allowed to stand for 10 minutes before being taken out of the glovebox. The Au mirror was washed with methanol (3 × 5 mL) followed by acetone (3 × 10 mL) and dried under an N_2_ stream. Method 1 was performed according to this protocol.

### Vacuum deposition at 120 °C

60 μL of a 10 mM methanolic solution of the NHC precursor was deposited onto the gold mirror and allowed to dry. Then, the mirror was heated at 120 °C under vacuum for 24 minutes. The resulting mirrors were then rinsed with reagent alcohol. Method 2 was performed according to this protocol using 1-CO_2_.

### Methanolic deposition at 55 °C

The electrochemically cleaned Au mirrors were immersed in a 10 mM methanolic solution of the NHC precursor and incubated for 24 hours at 55 °C. The Au mirrors were then rinsed with reagent alcohol. Method 3 was performed according to this protocol using (1-H)(HCO_3_).

### Methanolic deposition at 23 °C

The electrochemically cleaned Au mirrors were immersed in a 10 mM methanolic solution of the NHC precursor and incubated for 24 hours at room temperature. The Au mirrors were then rinsed with reagent alcohol. Method 4 and Method 5 were performed according to this protocol using (1-H)(HCO_3_) and (1-H)(OTf), respectively. The ambient temperature at the laboratory where samples for SERS, LDI-MS, and XPS were prepared was 23 °C, and the ambient temperature at the laboratory where samples for electrochemical characterization were prepared was 25 °C.

### Synthesis of 60 nm gold colloids

Gold nanoparticles were synthesized using a modified Frens^95,96^ method. Our groups have shown previously that this method produces gold colloids with an average size of 60 ± 10 nm (average ± std. dev.).^54^ Briefly, 120 microliters of 1% by weight trisodium citrate (Sigma Aldrich) was added to a boiling solution of 180 mL of 0.01% by weight gold(iii) chloride trihydrate (Sigma Aldrich) with vigorous stirring. Boiling continued for 30 minutes, then the suspension was allowed to cool to room temperature. Ultrapure water and aqua regia (caution, extremely dangerous) cleaned glassware were used for nanoparticle synthesis.

### Surface enhanced Raman spectroscopy (SERS)

20 microliters of citrate-capped gold nanoparticles were drop-cast onto the gold mirror and allowed to dry. SERS measurements were acquired on a custom-built Raman setup^43,51,91^ using a 633 nm HeNe laser (Thorlabs) and a laser power of 1 mW (measured at the objective using LaserCheck power meter by Coherent). The laser was focused on the sample using an inverted microscope (Nikon) with a 10× objective lens. The scattered light was collected through the same objective and then passed through a Rayleigh rejection filter (Semrock), which was then fed into the spectrometer (Princeton Instruments Acton). Scattered radiation was detected by a CCD detector (Princeton Instruments) cooled by liquid nitrogen. Six SERS measurements were collected per sample for three gold mirrors per deposition protocol for a total of 18 spectra, which are used to generate the standard deviation spectra shown in Fig. 2. Spectra were baseline subtracted, normalized to the most intense signal in the spectrum, then averaged together to generate the spectra shown in this work. The average of SERS data obtained for each method along with the corresponding blanks are shown in Fig. S1 and S3–S5.†

### Laser desorption/ionization mass spectrometry (LDI-MS)

Experiments were conducted using a Bruker UltrafleXtreme MALDI-TOF-TOF instrument equipped with a frequency tripled Nd:YAG laser (355 nm). Gold mirrors were immobilized on a polished steel sample target using copper tape and a custom PEEK sample adapter. All mass spectra were obtained in positive ion mode with the reflectron in operation (Fig. S2–S5†). The maximum laser power was expressed as a percentage relative to the highest achievable power with a global attenuator offset of, in general, 25%. Typically, a minimum of 2000 laser shots per spectrum were accumulated with a laser power set at 100% (maximum power). The instrument was calibrated using Au clusters according to the method of Havel.^97^

### X-Ray photoelectron spectroscopy

For XPS analysis of gold film samples, a PHI VersaProbe II surface analysis instrument from Physical Electronics (Chanhassen, MN) equipped with a monochromatic Al Kα X-ray source (photon energy = 1486.6 eV) was used. Five different locations on the substrate were probed to acquire high-resolution spectra using a 23.50 eV pass energy with ultra-high vacuum conditions. For the survey, N 1s, and Au 4f (data not shown) regions, we collected 7, 30, and 20 sweeps respectively (Fig. S8–S17†). The resulting spectra were summed together after calibration to the Au 4f peak at 83.98 eV,^98^ then the summed spectra were also calibrated to the Au 4f peak at 83.98 eV. XPS spectra were processed using a linear^99^ Shirley,^100^ or Tougaard^101^ background subtraction and peak fitting was performed using a Voigt peak shape in CasaXPS.^100^ The handbook of X-ray photoelectron spectroscopy,^98^ CasaXPS software, and previous studies of NHC monolayers on gold^58^ were used to interpret XPS data. During peak fitting, the N 1s full-width half maximum values were restricted to a maximum of 2 eV according to the method of Crudden and McLean.^58^

### General synthetic considerations

The syntheses of 1,3-diisopropyl benzimidazolium bromide, (1-H)(Br),^102^ and 1,3-diisopropyl benzimidazolium hexafluorophosphate, (1-H)(PF_6_),^103^ were carried out according to previously published procedures. Solution state ^1^H, ^13^C{^1^H}, ^31^P{^1^H}, and ^19^F{^1^H} NMR experiments were performed on Bruker Avance 500 MHz narrow-bore broadband system at 298 K (Fig. S18–S25 and S29†). The ^1^H NMR spectral peaks were referenced to the residual protonated solvents. The ^13^C{^1^H} NMR spectral peaks were referenced to the solvents. The ^31^P{^1^H} and ^19^F{^1^H} NMR spectral peaks were referenced to the external standards of 85% H_3_PO_4_ and CF_3_COOH respectively. 1-CO_2_^42,43^ and (1-H)(OTf)^70^ were synthesized according to our groups’ previously reported protocols. Single crystal X-ray diffraction (SCXRD) analysis of (1-H)(HCO_3_) was performed using Bruker D8 Venture Diffractometer at 100 K with CuKα radiation at the University of Tennessee.

Additional studies and new characterization were collected on (1-H)(HCO_3_). A solid state structure of (1-H)(HCO_3_) was successfully obtained using single crystal X-ray diffraction (SCXRD) (Fig. S28 and Table S1†). The structure confirmed the existence of bicarbonate anion with the presence of seven water molecules per asymmetric unit.

The counteranion influences the chemical shift value of the ^1^H signal on the 2-position (*i.e.* the carbene carbon).^103^ Because the exact position of this resonance is typically diagnostic for anion confirmation, we conducted ^1^H NMR experiments of (1-H)(HCO_3_) in DMSO-*d*_6_ at different concentrations of (1-H)(HCO_3_) (Fig. S29†). The results show that the ^1^H signal of the CH on the carbene carbon shifted as the concentration of (1-H)(HCO_3_) changed in DMSO-*d*_6_. This shift is due to the fact that bicarbonate anion is a base and strong hydrogen bond acceptor. Therefore, increasing the concentration of (1-H)(HCO_3_) polarizes the H-atom on the carbene carbon and increases the acidity of the (1-H) salt, resulting in downfield shifts in the ^1^H NMR signals.

### Synthetic procedures for 1,3-diisopropylbenzimidazolium bicarbonate (1-H)(HCO_3_)

Preparation of the precursor was performed from a reported procedure.^62^ All synthetic reactions to prepare (1-H)(HCO_3_) were conducted under air unless otherwise stated. Solvents were used without purification except where stated. Unless otherwise noted, chemicals were purchased from chemical suppliers used as received. Amberlyst A26 hydroxide resin was activated by sparging a solution with CO_2_ for 30 minutes before use as the HCO_3_ resin. ^1^H and ^13^C{^1^H} NMR (Fig. S26 and S27†) spectra were recorded at Queen's University using Bruker Avance 400, 500, or 700 MHz spectrometers at 298 K. Chemical shifts (*δ*) are reported in parts per million (ppm) and are referenced to residual protonated (^1^H) or deuterated (^13^C{^1^H}) solvent signals. Coupling constants (*J*) are reported as absolute values. All NMR data were processed and displayed using Bruker TopSpin or MestReNova softwares. Elemental analyses were performed at Queen's University using Flash 2000 CHNS-O analyzer.

### 1,3-Diisopropylbenzimidazolium bicarbonate

1,3-Diisopropylbenzimidazolium bicarbonate was prepared from previously reported procedures.^62^ Resin-HCO_3_ (250 mL) suspended in water was measured in a graduated cylinder and transferred to a scintillation vial where the resin was allowed to settle, and water was decanted off. The resin was washed with methanol three times. 1,3-Diisopropylbenzimidazolium iodide (16.5 g, 1 eq., 50 mmol) was dissolved in methanol and transferred to the resin. The mixture was stirred for 1 hour. The solution was passed through a cotton plug and washed with methanol. The solvent was evaporated in a stream of air and the crude oily product was triturated in acetone and diethyl ether (1 : 1). The solvent was decanted, and trituration was repeated. Subsequent drying under high vacuum afforded the product as a white powder (57% yield, 7.56 g). ^**1**^**H NMR** (700 MHz, CD_3_OD) *δ* 8.07–8.02 (m, 1H), 7.75–7.70 (m, 1H), 5.08 (sept, *J* = 6.7 Hz, 1H), 1.75 (d, *J* = 6.8 Hz, 6H). ^**13**^**C NMR** (CDCl_3_, 174 MHz): *δ* 161.42, 138.96, 132.59, 128.19, 114.97, 52.83, 22.11 **CHN**: calculated for C_14_N_2_O_3_H_20_: C: 63.62, H: 7.63, N: 10.60 found: C: 62.41, H: 7.67, N: 10.31.

### Electrochemical characterization of gold electrodes – chemicals and materials

Phosphate buffered saline (1× PBS, 11.9 mM HPO_4_^2−^; 137 mM NaCl; 2.7 mM KCl; pH = 7.4), and sulfuric acid (H_2_SO_4_), were purchased from Fisher-Scientific (Waltham, MA). All aqueous solutions were prepared using deionized water from a Milli-Q Direct purification system, with a resistivity of 18 MΩ. Gold disk electrodes (PN 002314, diameter 1.6 mm) and coiled platinum wire counter electrodes (PN 012961) were purchased from ALS Inc. (Tokyo, Japan). Silver/silver chloride reference electrodes (PN CHI111) were purchased from CH instruments (Austin, TX). For polishing electrodes 0.1 μM diamond slurry was purchased from Buehler (Lake Bluff, IL) and cloth pads (PN MF-1040) were purchased from BASi (West Lafayette, IN).

### Electrochemical characterization of gold electrodes – electrochemical measurements

CH Instruments Electrochemical Analyzer (CHI 1040C, Austin, TX) multichannel potentiostats and associated software were used for all electrochemical measurements. A three-electrode cell configuration consisting of a working electrode, a coiled platinum wire counter electrode, and an Ag/AgCl reference electrode was used. For the continuous cyclic voltammetric interrogation a voltage window beginning at 0 V sweeping to 0.3 V, returning to −0.1 V, and concluding at 0 V for 3 total sweep segments with a scanning rate of 0.1 V s^−1^ after a quiet time of 2 s.

### Electrochemical characterization of gold electrodes – data analysis

To process the files generated during the continuous voltametric interrogation, we used a Python-based custom script previously reported by our group (SACMES).^104^ SACMES allows for the rapid extraction of capacitive currents from voltammograms, thus enabling batch analysis of the thousands of files generated during measurements.

### Electrochemical characterization of gold electrodes – preparation of NHC functionalized electrodes

Electrodes were first cleaned by a 30 seconds placement into a thiourea based gold surface cleaning solution (Mettler Toledo, Leicester, UK) with constant swirling. We then polished the gold electrodes on a cloth pad with a 0.1 μM diameter diamond slurry for 2 min per electrode. Electrodes were then placed in a beaker of PBS and cycled using cyclic voltammetry from −0.1 V to –0.3 V (*vs.* Ag|AgCl) to confirm removal of NHCs with a return to bare gold baseline voltammograms. Following PBS scans, electrodes were electrochemically roughened *via* chronoamperometry to increase electroactive surface area following previously published protocols.^105^ In short, 320 pulses (0.01 s pulse width) from 0 to 2 V (*vs.* Ag|AgCl), was repeated 50 times for a total of 16 000 pulses in 0.5 M H_2_SO_4_. Immediately following roughening, electrodes were placed in 0.05 M H_2_SO_4_ and cycled once to ascertain the active surface area of each electrode. After surface area determination, electrodes were functionalized with NHCs according to previously described procedures with slight modifications described in the following paragraphs. In each case, 10 microliters of NHC stock solution was drop cast onto the electrode surface.

For oven deposition, electrodes were placed upright in a beaker and 10 microliters of a 10 mM NHC methanol solution was drop casted on the electrode surface and allowed to evaporate for 10 min. Electrodes were then wrapped in aluminum foil and placed in a vacuum oven (Thermo Scientific, 3618-1CE) at 120 °C, 10 inches of mercury (inHg) (30 inHg room pressure, vacuumed to −20 inHg) for 25 min. Electrodes were then allowed to cool slowly to room temperature on the benchtop prior to electrochemical characterization. To reduce contamination, the oven was left under constant heat and pressure, and the aluminum foil was discarded after every functionalization. Since the gold electrodes (1.6 mm diameter) used for electrochemistry studies have a smaller surface are than the gold mirrors (approximately 12 by 12 mm) used for SERS, LDI-MS, and XPS measurements, the volume of the NHC stock solution was reduced for electrochemistry experiments to account for the reduced surface area.

For the room temperature deposition, electrodes were placed in a solution of 10 mM NHC in methanol in Eppendorf tubes. Tubes were sealed with parafilm and placed in the dark for 24 hours.

For 55 °C deposition, electrodes were placed in a solution of 10 mM NHC in methanol in Eppendorf tubes. Tubes were sealed with parafilm and placed on an Eppendorf Thermomixer (PN: P 5382000023) at 55 °C for 24 hours.

## Author contributions

A. C. prepared NHC Au mirror samples, characterized samples with SERS and LDI-MS, analyzed data, and wrote the first draft of the manuscript. N. L. D. analyzed data and conducted XPS experiments. G. K. synthesized NHC compounds and performed the free carbene deposition. V. C. performed electrochemical characterization of NHC systems. P. N., I. M. J., and M. D. A. synthesized and characterized NHC compounds. L. C. E. assisted with sample preparation, XPS, and LDI-MS data collection. N. L. D., D. M. J., and J. P. C. collaboratively designed the experiments and supervised the project. C. M. C., N. A. C., D. M. J., and J. P. C. all edited the manuscript and contributed to the data interpretation. All authors contributed to the writing and editing of the final manuscript.

## Data availability

The data supporting this study are available from the corresponding authors upon reasonable request.

## Conflicts of interest

There are no conflicts to declare.

## Supplementary Material

NR-017-D4NR04428B-s001

NR-017-D4NR04428B-s002
