# Supplementary material for: Forming N-heterocyclic carbene monolayers: not all deposition methods are the same
Source: Nanoscale. 2025 Jan 24;17(9):5413–28. doi: 10.1039/d4nr04428b (PMC11788998; doi:10.1039/d4nr04428b)
Supplement: NR-017-D4NR04428B-s001 [file NR-017-D4NR04428B-s001.pdf]

Supplementary Information (SI) for Nanoscale.
This journal is © The Royal Society of Chemistry 2025

Supporting Information for "Forming N-Heterocyclic Carbene Monolayers: Not All Deposition Methods are the Same"

Aruna Chandran,[1,‡] Nathaniel L. Dominique,[1,‡] Gurkiran Kaur,[2] Vincent Clark,[3] Phattananawee Nalaoh,[2] Lilian Chinenye Ekowo,[1] Isabel M. Jensen,[2] Mark D. Aloisio,[4,5] Cathleen M. Crudden,[4,5,6] Netzahualcóyotl Arroyo Currás,[3,7] David M. Jenkins,[2]* Jon P. Camden[1]*

1 Department of Chemistry and Biochemistry, University of Notre Dame, Notre Dame, IN 46556, United States
2 Department of Chemistry, University of Tennessee, Knoxville, TN 37996, United States
3 Chemistry-Biology Interface Program, Zanvyl Krieger School of Arts & Sciences, Johns Hopkins University, Baltimore, MD 21218, United States
4 Department of Chemistry, Queen's University, 90 Bader Lane, Kingston, Ontario K7L 3N6, Canada
5 Carbon to Metal Coating Institute, C2MCI, Queen's University, 90 Bader Lane, Kingston, Ontario K7L 3N6, Canada
6 Institute of Transformative Bio-Molecules (WPI-ITbM), Nagoya University, Nagoya 464-8601, Japan
7 Department of Pharmacology and Molecular Sciences, Johns Hopkins University School of Medicine, Baltimore, MD 21205, United States

***Correspondence to:**
Jon P. Camden
Department of Chemistry and Biochemistry
University of Notre Dame
Notre Dame, Indiana 46556
jon.camden@nd.edu

David M. Jenkins
Department of Chemistry
University of Tennessee, Knoxville
Knoxville, TN 37996
jenkins@ion.chem.utk.edu

‡ Aruna Chandran and Nathaniel L. Dominique are equally contributing first authors.

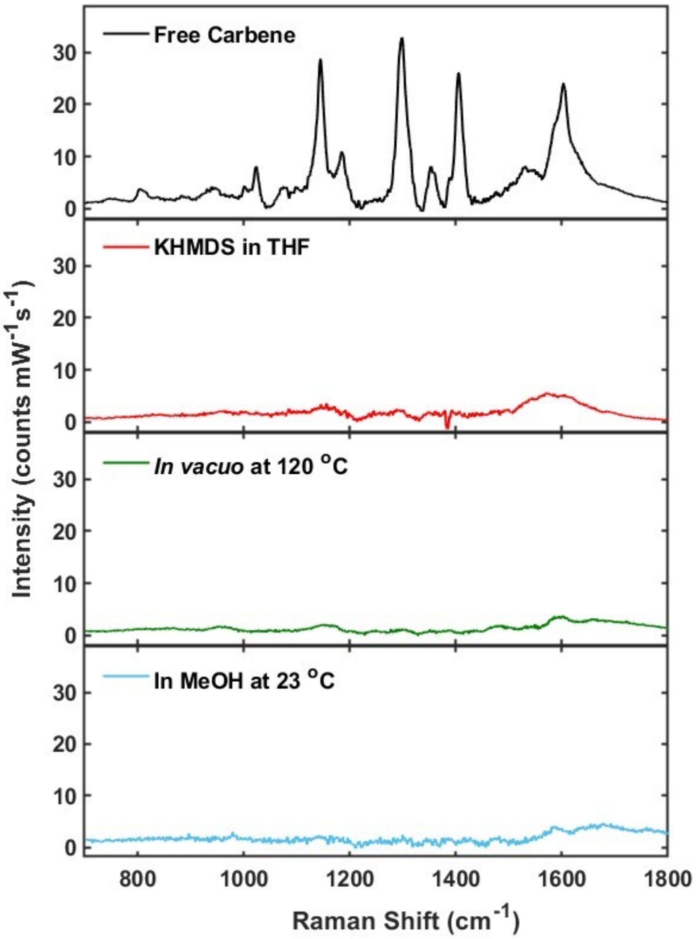

Figure S1. SERS of Au Mirrors Subjected to Deposition Conditions without NHC Precursors. SER spectra of an Au mirror exposed to a solution of KHMDS in THF without adding NHC precursor (red). SER spectra of Au mirror heated at 120 °C *in vacuo* for 24 minutes (green). SER spectra of Au mirror incubated in methanol without any NHC precursors (blue) for 24 hours. No SERS signatures with a comparable signal intensity to the benchmark free carbene method (black) were observed for any controls.

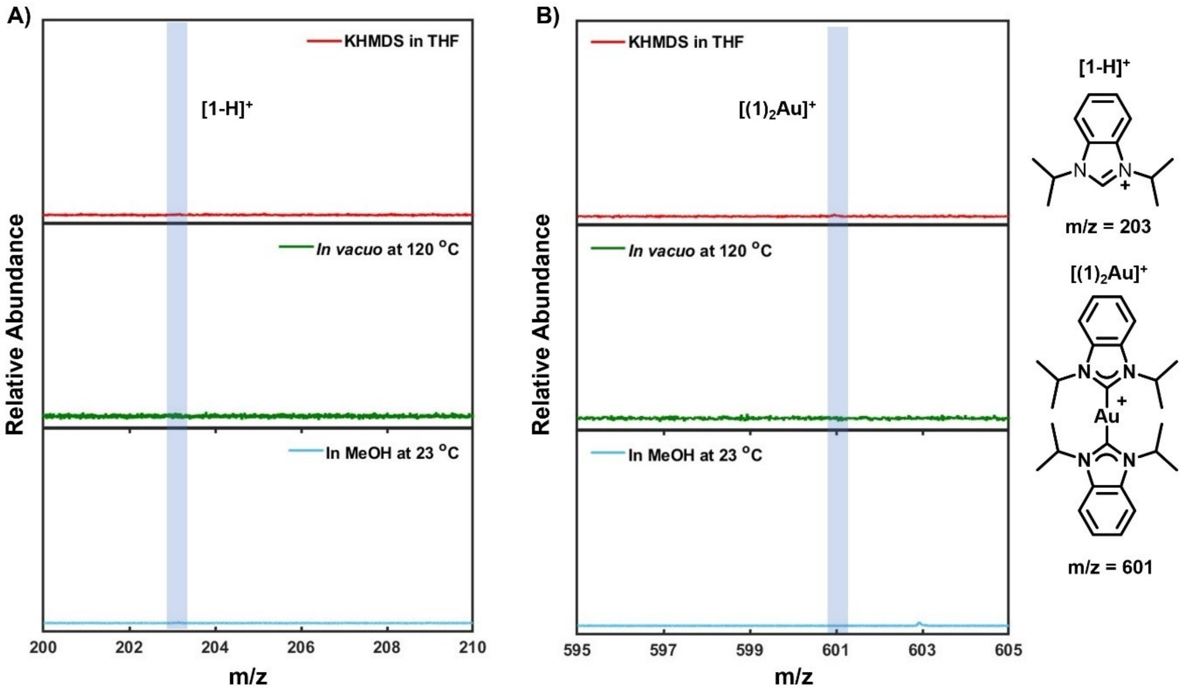

Figure S2. LDI-MS of Au Mirrors Subjected to Deposition Conditions without NHC Precursors. LDI-MS spectra of gold mirrors subjected to the free carbene, vacuum, and methanolic deposition methods without NHC precursors. The LDI-MS spectra are shown for the [**1**-H]$^+$ ion channel (A) and the [(**1**)$_2$Au]$^+$ ion channel (B).

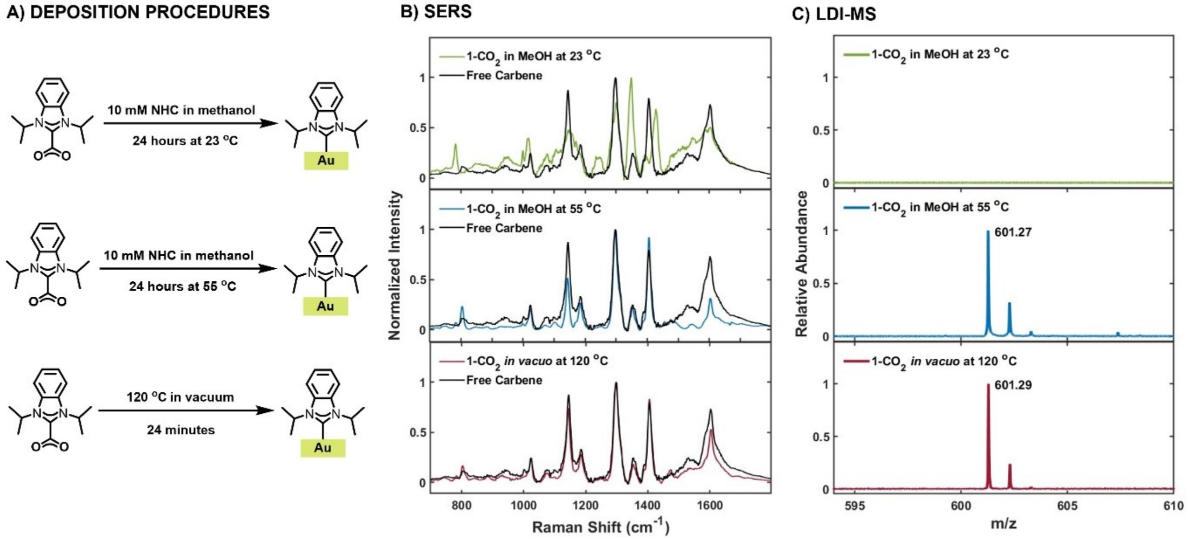

Figure S3. SERS and LDI-MS of Au Mirrors Treated with 1-CO_2. (A) Deposition procedures starting with **1**-CO_2. (B) SERS and (C) LDI-MS data of Au mirrors treated with **1**-CO_2 *via* room temperature methanolic deposition (green), heated methanolic deposition (blue), and the heated deposition *in vacuo* (red). The SER spectrum of the free carbene deposition is overlayed for comparison (black). SERS and LDI-MS data is in excellent agreement with an NHC bound to gold with the exception of the room temperature methanolic deposition. In this case, the SERS signatures differ dramatically from the free carbene benchmark method and no evidence of NHC-Au bond formation was observed in LDI-MS (i.e. $[(\mathbf{1})_2Au]^+$ ion at 601 *m/z*).

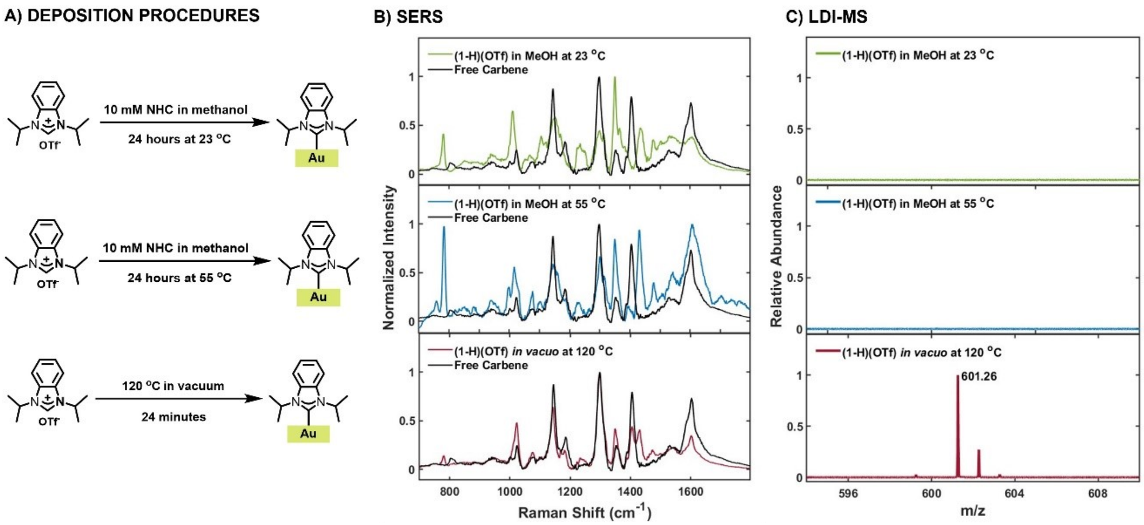

Figure S4. SERS and LDI-MS of Au Mirrors Treated with the (1-H)(OTf). (A) Deposition procedures starting with (**1**-H)(OTf). (B) SERS and (C) LDI-MS data of Au mirrors treated with (**1**-H)(OTf) via room temperature methanolic deposition (green), heated methanolic deposition (blue), and the heated deposition *in vacuo* (red). The SER spectrum of the free carbene deposition is overlayed for comparison (black). The SER spectra for all triflate depositions differ dramatically from the benchmark free carbene deposition, which illustrates that these triflate procedures produce different surface structures than the benchmark. However, under vacuum annealing conditions, both the SER spectra and the observation of the $[(\mathbf{1})_2Au]^+$ ion at 601 m/z in the LDI-MS suggest that the triflate precursor does produce legitimate overlayers, but only under these specific conditions.

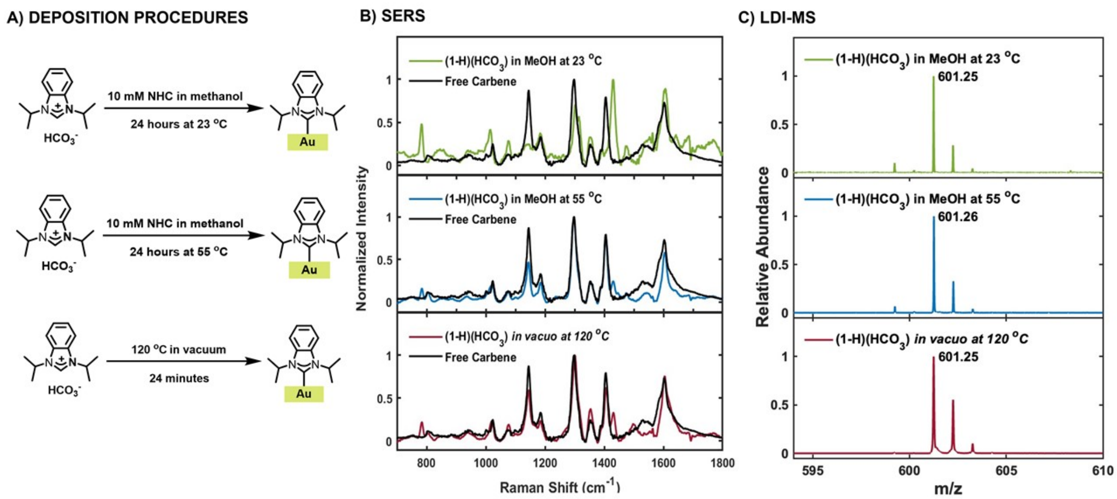

Figure S5. SERS and LDI-MS of Au Mirrors Treated with (1-H)(HCO_3). (A) Deposition procedures starting with (**1**-H)(HCO_3). (B) SERS and (C) LDI-MS data of Au mirrors treated with (**1**-H)(HCO_3) *via* the room temperature methanolic deposition (green), heated methanolic deposition (blue), and the heated deposition *in vacuo* (red). The SER spectrum of the free carbene deposition is overlayed for comparison (black). All SERS and LDI-MS data are in excellent agreement with an NHC bound to gold with the exception of the room temperature methanolic deposition. In this case, the SER spectra differ significantly from the benchmark free carbene method.

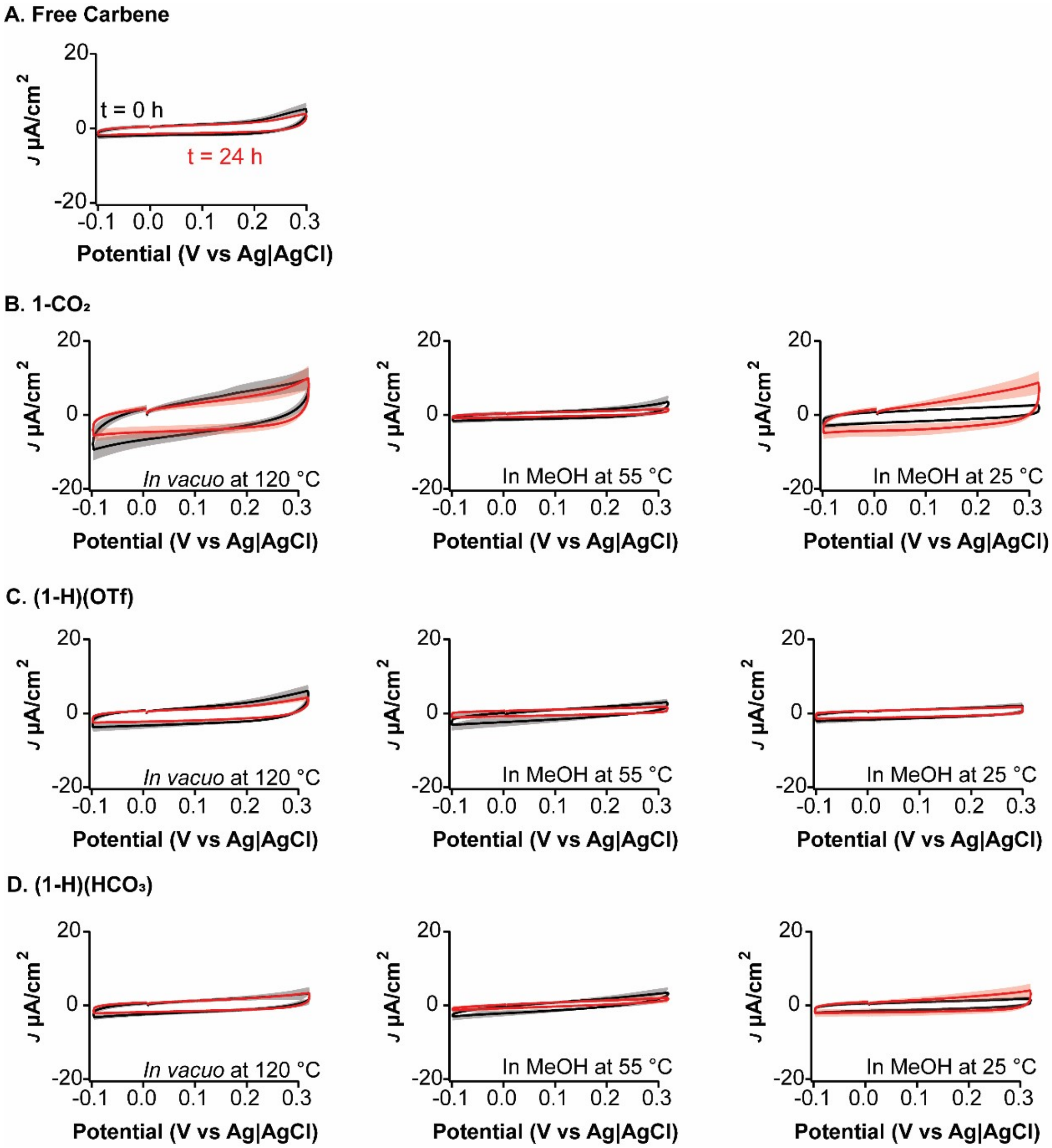

Figure S6. Cyclic Voltammograms of Au Electrodes Treated with NHC ligands. (A) The benchmark free carbene displays low initial capacitive currents (black trace, t = 0 hour) and remains unchanged even after 24 hours of continuous voltammetric cycling (t = 24 hours, red trace). These results indicate extensive electrode passivation and good electrochemical stability even after extensive cycling. For all gold electrodes treated with (**1**-H)(OTf) (B), **1**-CO_2 (C), or (**1**-H)(HCO_3) (D), comparable capacitive currents to the benchmark free carbene method were observed except for select deposition methods with **1**-CO_2. For the deposition of **1**-CO_2 *in vacuo* at 120 °C and in methanol at 25 °C, larger capacitive currents and decreased electrochemical stability were observed relative to the free carbene method. The solid lines and shaded regions represent the average and standard deviation of at least 4 samples, respectively. All capacitive currents were sampled at 0.1 V, using a voltage scanning rate of 100 mV s^{-1}.

A)

NHC Precursor	Deposition Method	i_c (μA/cm²) t = 0 h	i_c (μA/cm²) t = 24 h
(**1**-H)(OTf)	*In vacuo* at 120 °C	4.79 ± 1.5	3.1 ± 0.6
(**1**-H)(OTf)	In methanol at 25 °C	2.1 ± 0.9	1.4 ± 0.6
(**1**-H)(OTf)	In methanol at 55 °C	3.6 ± 1.3	1.7 ± 0.3
**1**-CO_2	*In vacuo* at 120 °C	13.7 ± 7.7	7.7 ± 3.1
**1**-CO_2	In methanol at 25 °C	3.5 ± 0.6	6.9 ± 2.4
**1**-CO_2	In methanol at 55 °C	2.3 ± 0.8	1.3 ± 0.2
(**1**-H)(HCO_3)	*In vacuo* at 120 °C	3.6 ± 2.2	2.4 ± 1.1
(**1**-H)(HCO_3)	In methanol at 25 °C	2.4 ± 0.6	3.2 ± 1.6
(**1**-H)(HCO_3)	In methanol at 55 °C	3.5 ± 1.3	1.5 ± 0.3
(**1**-H)(PF_6)	Free Carbene	2.7 ± 1.1	2.1 ± 0.8

B)

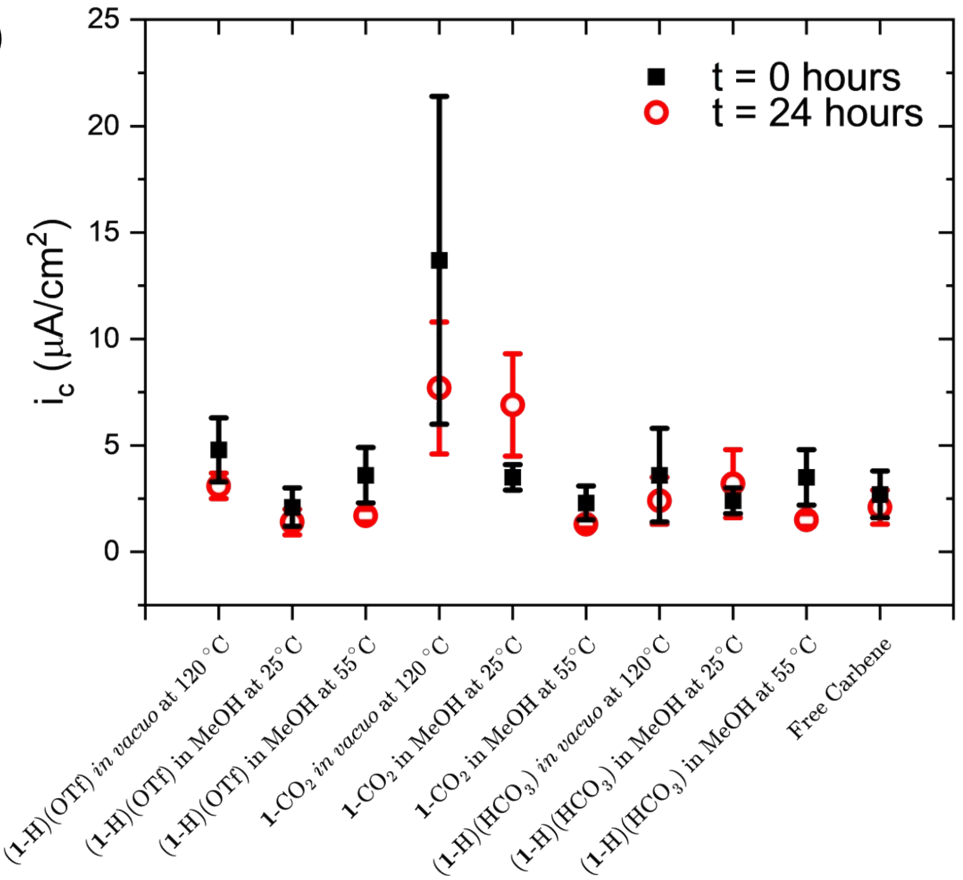

Figure S7. Capacitive Current Measurements for Au Electrodes Treated with NHC Ligands. (A) Table presenting the capacitive current at 0.1 V at t = 0 hour and after continuous electrochemical cycling of the NHC-Au electrode (t = 24 hours). (B) The capacitive current measurements with standard deviation at t = 0 hour (black) and t = 24 hours (red). As Figure S6 illustrates, the deposition of **1**-CO_2 *in vacuo* at 120 °C and in methanol at 25 °C produces gold surfaces with higher capacitive currents and reduced electrochemical stability relative to the other methods.

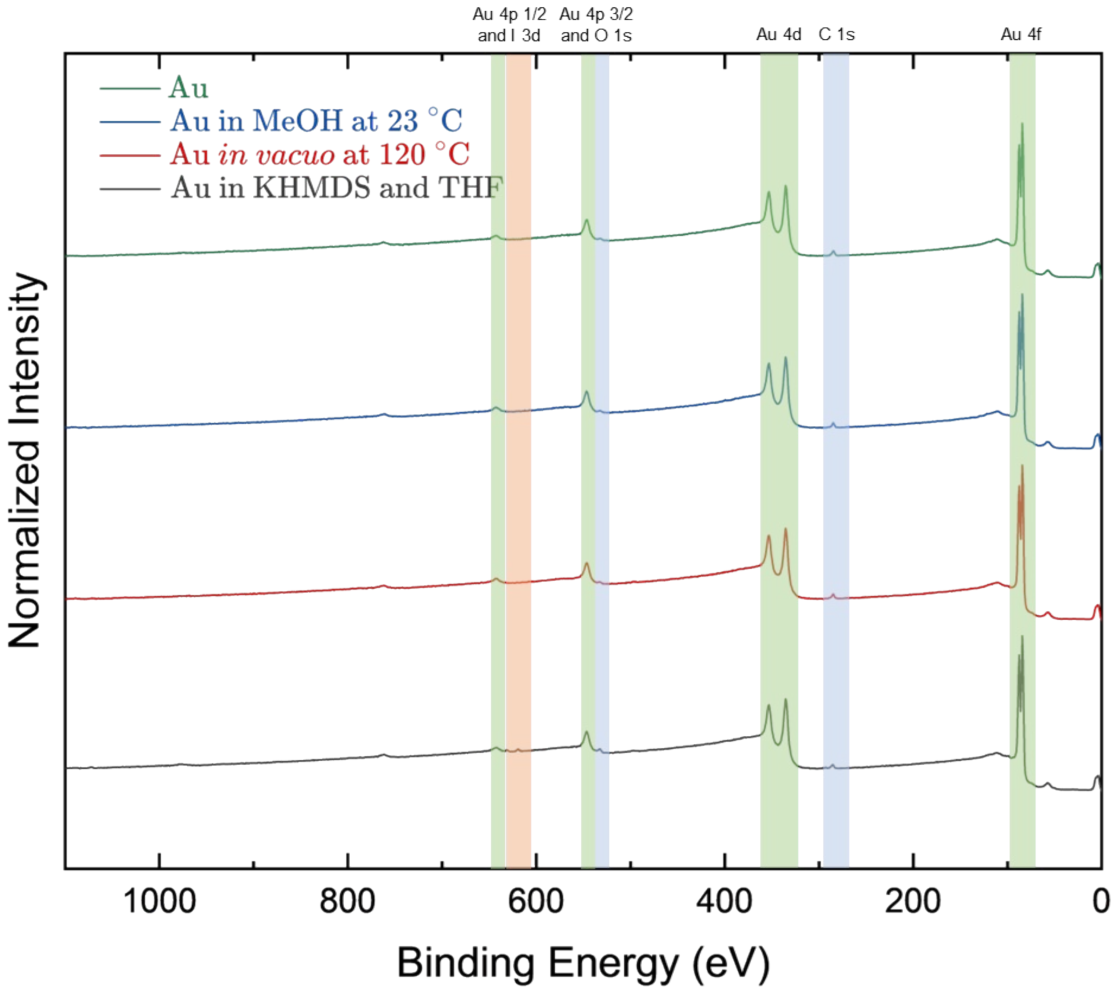

Figure S8. XPS Survey Scans of Au Mirrors Subjected to Deposition Conditions without the NHC Ligands. XP spectra for the Au mirror (green), Au mirror soaked in HPLC grade methanol for 24 hours at room temperature (blue), Au mirror *in vacuo* at 120 °C for 24 minutes (red), and Au mirror treated with KHMDS in THF (black). The C 1s peaks in the spectra of Au mirror blanks arise from adventitious carbon, which forms on surfaces exposed to atmospheric conditions as is well documented in XPS.[1-4] Trace amounts of iodine are present on the surface after exposure to KHMDS in THF, illustrating that trace iodine impurities are present in commercial KHMDS samples.

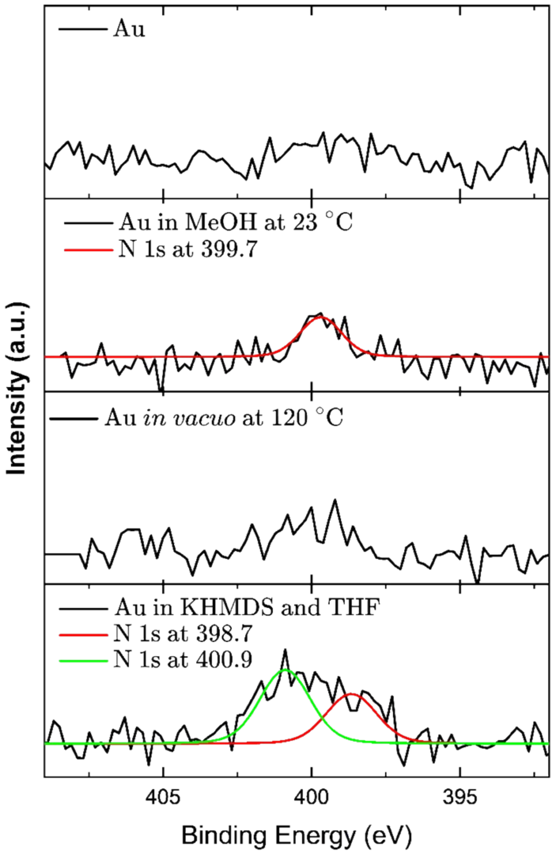

Figure S9. XPS N 1s Elemental Scans of Au Mirrors Subjected to Deposition Conditions without the NHC Ligands. The presence of a nitrogen peak in the N 1s region of Au mirrors treated with methanol illustrates that there may be a nitrogen containing impurity in the HPLC-grade methanol. Trace organic impurities in methanol have been reported in the literature[5-7] and may give rise to nitrogen containing surface contamination. N 1s signals observed for the Au mirror treated with KHMDS in THF (bottom spectrum) are attributed to residual KHMDS.

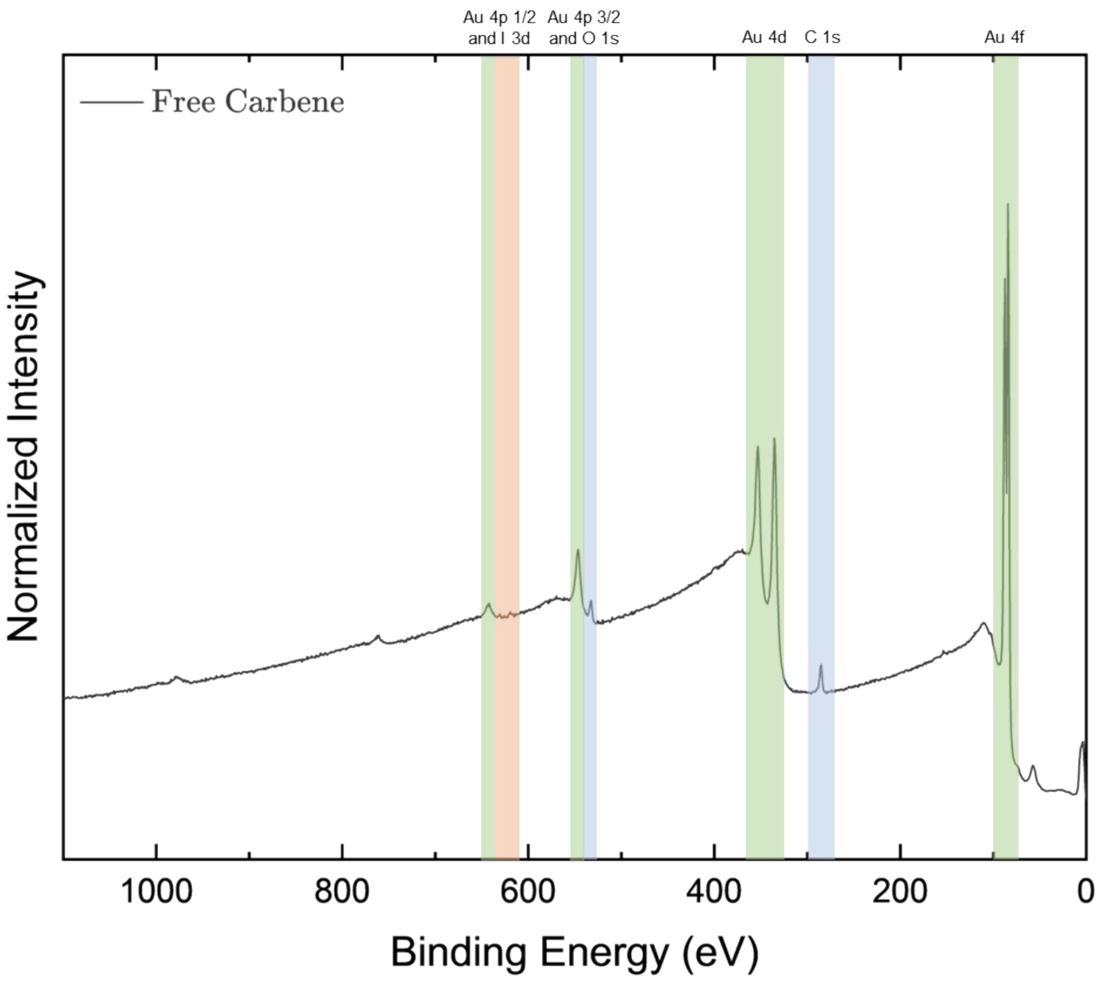

Figure S10. XPS Survey Scan of Au Mirrors Prepared via the Free Carbene Method. Iodine was detected as a trace surface contaminant, which has been observed in previous studies of NHCs on gold.[8]

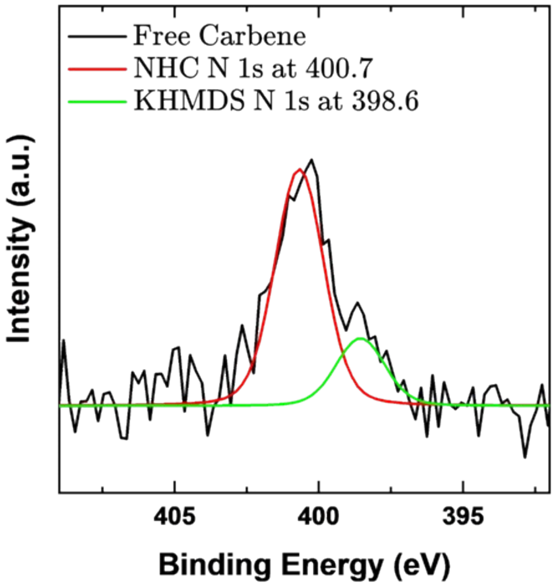

Figure S11. XPS N 1s Elemental Scan of Au Mirrors Prepared via the Free Carbene Method. The presence of a nitrogen peak in the N 1s region at 398.6 eV arises from residual KHMDS. The N 1s signal from the free carbene is 2x more intense than control signals and is in agreement with the N 1s peak position for NHC-Au systems between 399.9 and 401.0 eV.[9-12]

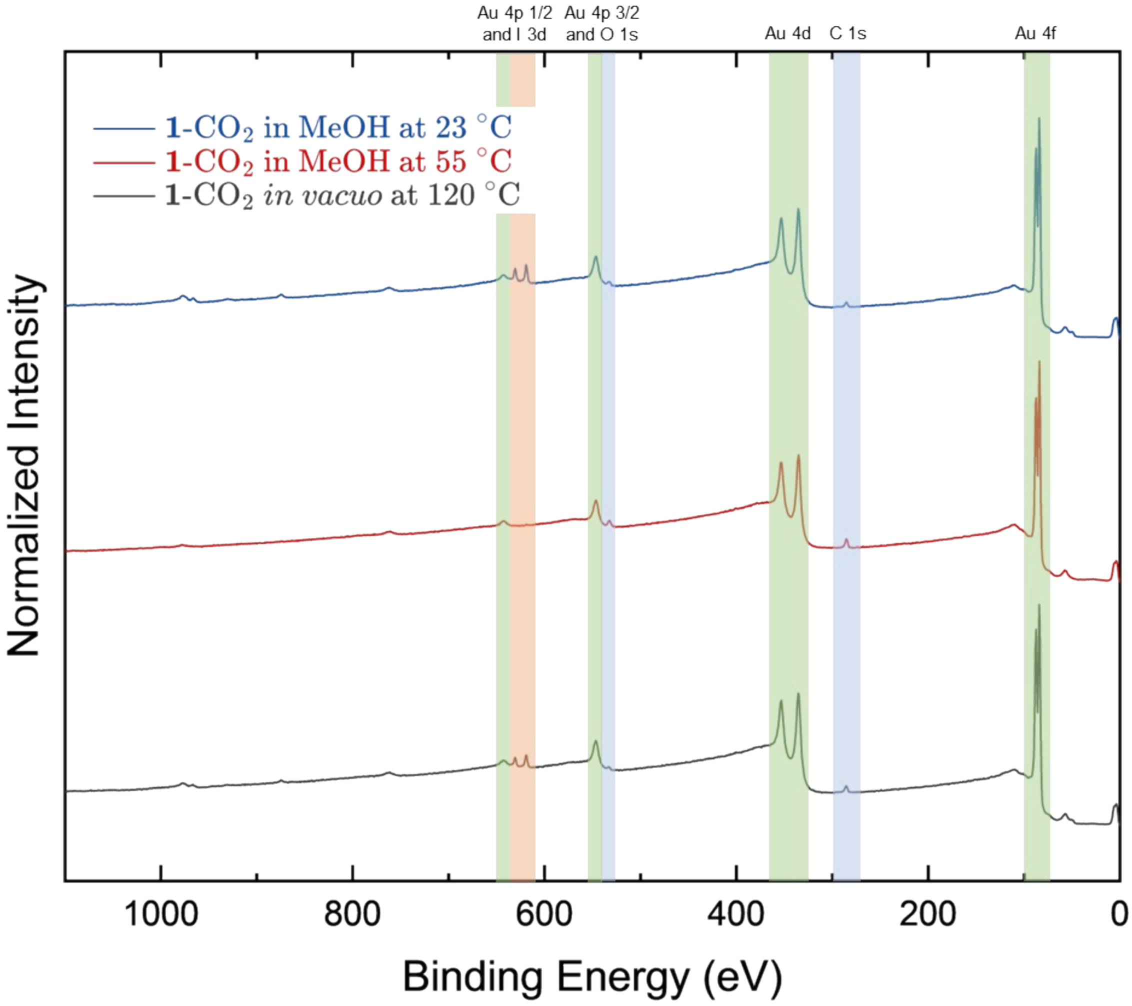

Figure S12. XPS Survey Scan of Au Mirrors Treated with 1-CO_2. Au mirrors treated with **1**-CO_2 via room temperature methanolic deposition (blue), heated methanolic deposition at 55 °C (red), and *in vacuo* deposition at 120 °C (black). Iodine was observed in the XPS spectra, which has been observed in previous studies of NHC monolayers on gold.[8]

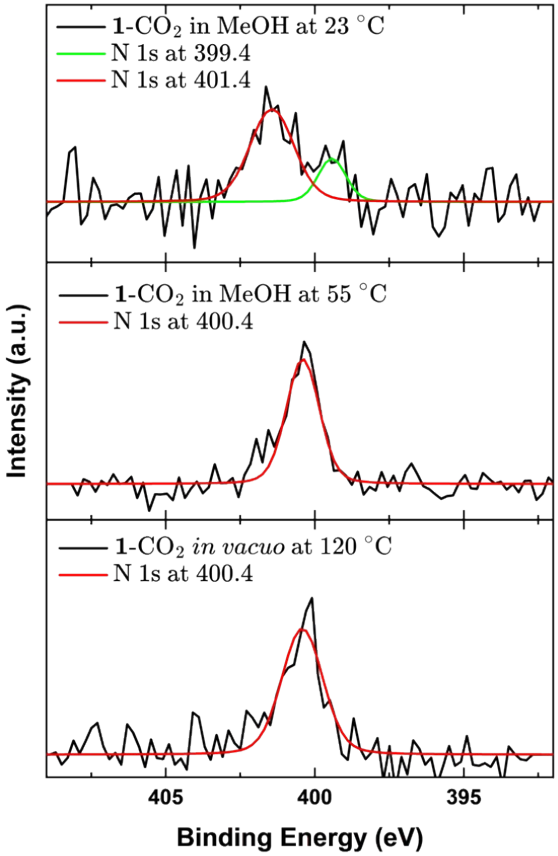

Figure S13. XPS N 1s Elemental Scans of Au Mirrors Treated with 1-CO_2. Au mirrors treated with **1**-CO_2 via room temperature methanolic deposition (top), heated methanolic deposition at 55 °C (middle), and *in vacuo* deposition at 120 °C (bottom). For room temperature methanolic deposition, the N 1s signal at 399.4 eV is approximately the same intensity as the peak arising from a nitrogen containing impurity observed in the control experiment (Figure S9). This feature in the spectra therefore may arise due to the impurity. The other N 1s signal at 401.4 eV is in agreement with previously reported binding energies for unbound NHC ligands.[12-14] The heated methanolic deposition and vacuum deposition conditions are in agreement with the N 1s peak position for NHC-Au systems between 399.9 and 401.0 eV.[9-12]

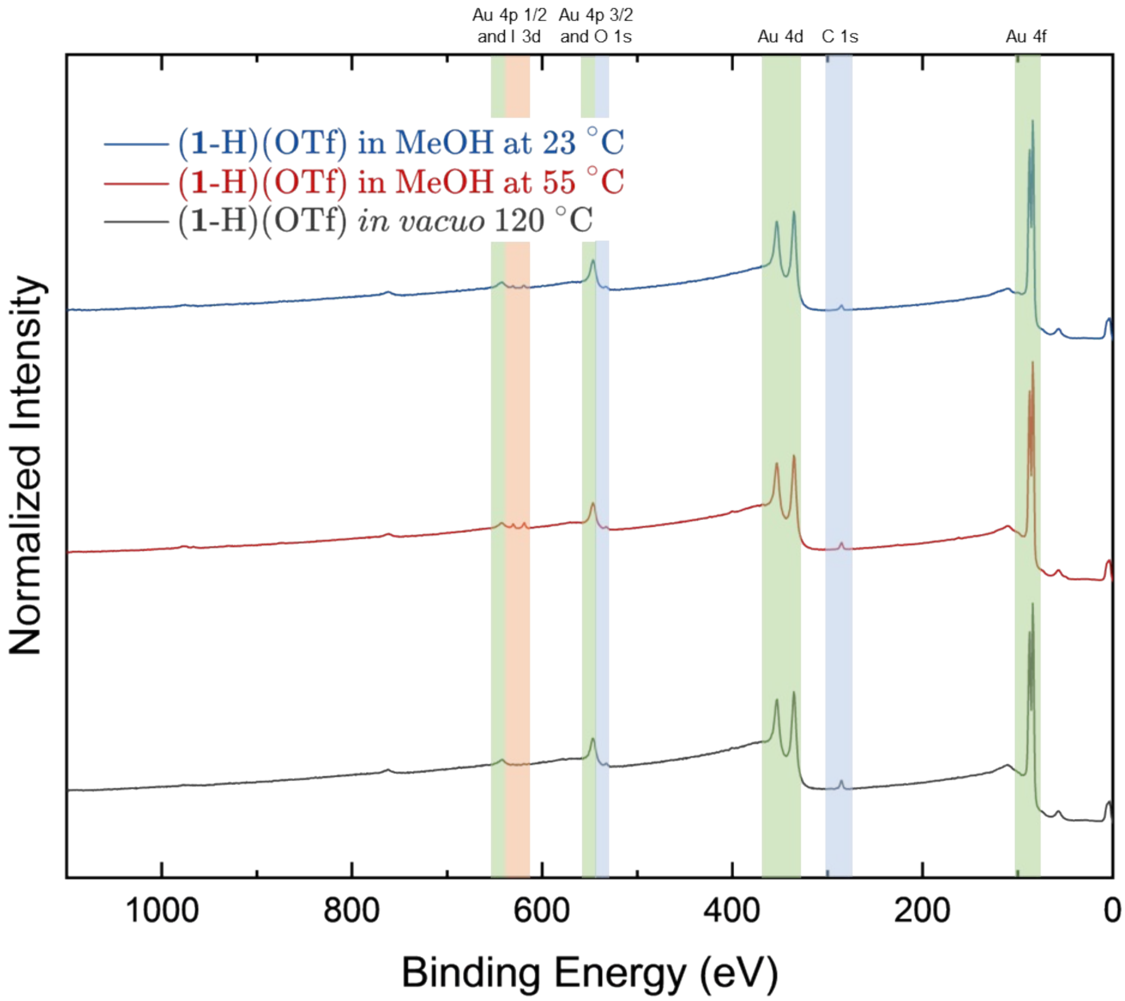

Figure S14. XPS Survey Scan of Au Mirrors Treated with (1-H)(OTf). Au mirrors treated with (**1**-H)(OTf) via room temperature methanolic deposition (blue), heated methanolic deposition at 55 °C (red), and *in vacuo* deposition at 120 °C (black). Iodine was observed in the XPS spectra, which has been observed in previous studies of NHC monolayers on gold.[8]

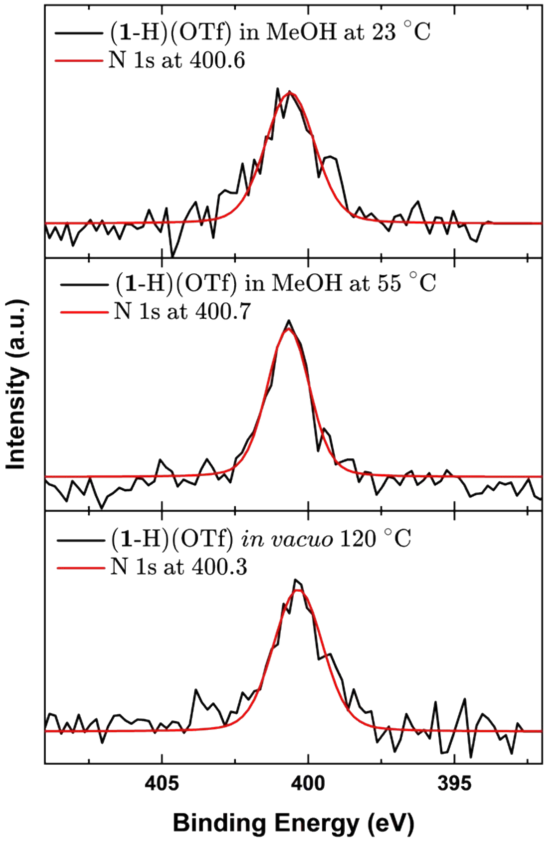

Figure S15. XPS N 1s Elemental Scans of Au Mirrors Treated with (1-H)(OTf). Au mirrors treated with (**1**-H)(OTf) via room temperature methanolic deposition (top), heated methanolic deposition at 55 °C (middle), and *in vacuo* deposition at 120 °C (bottom). The N 1s peak position for NHC-Au systems prepared via all three methods is within the literature range for NHC bound gold surfaces (399.9 and 401.0 eV).[9-12]

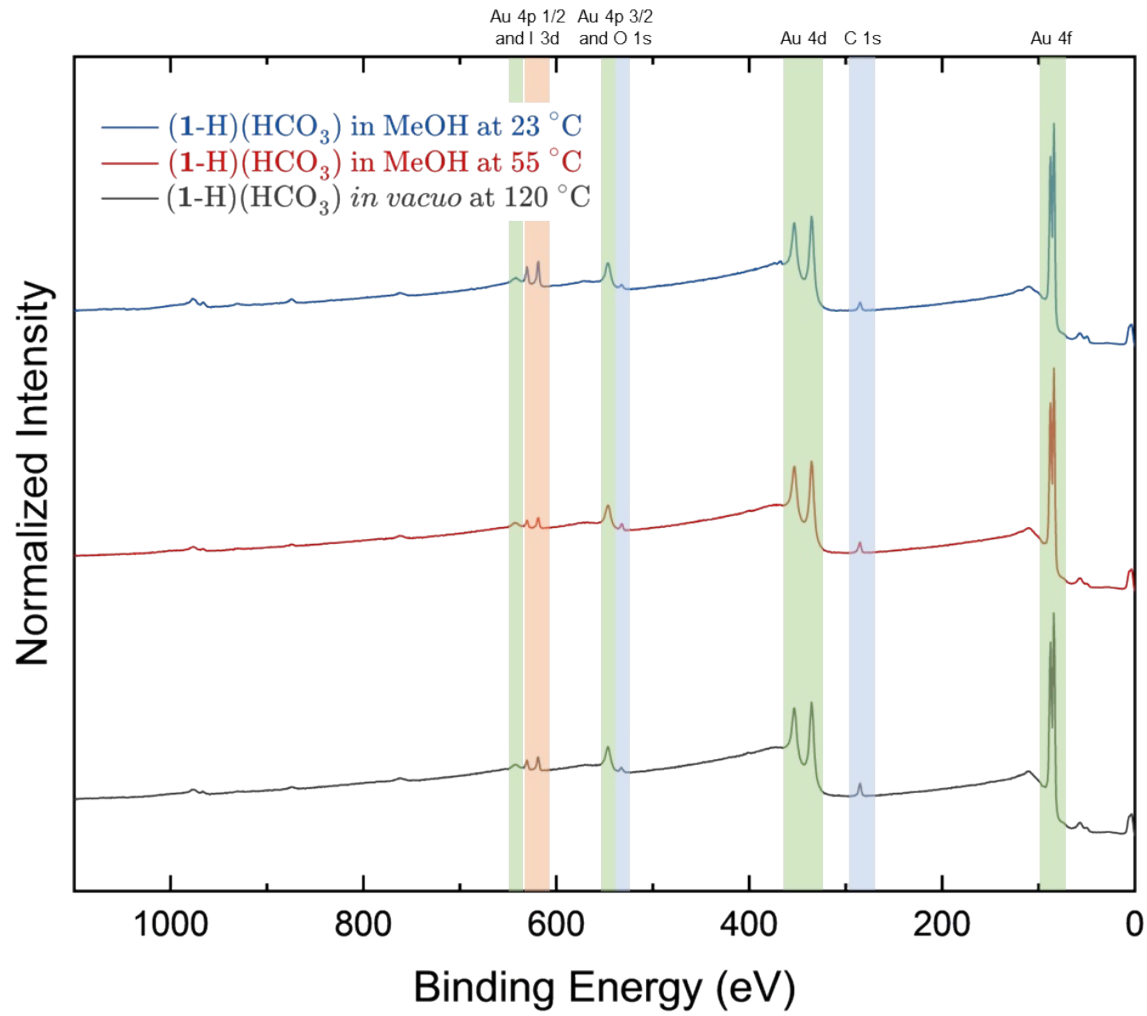

Figure S16. XPS Survey Scan of Au Mirrors Treated with (1-H)(HCO_3). Au mirrors treated with (**1**-H)(HCO_3) via room temperature methanolic deposition (blue), heated methanolic deposition at 55 °C (red), and *in vacuo* deposition at 120 °C (black). Iodine was observed in the XPS spectra, which has been observed in previous studies of NHC monolayers on gold.[8]

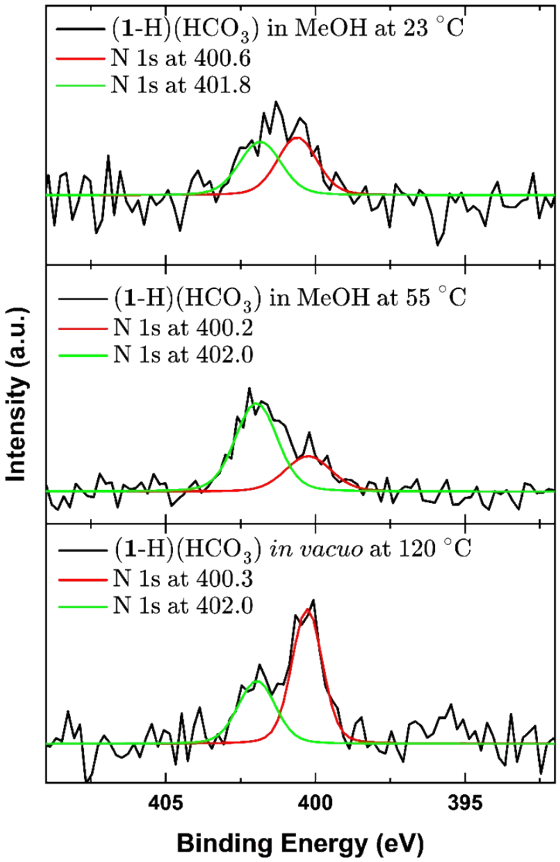

Figure S17. XPS N 1s Elemental Scans of Au Mirrors Treated with (1-H)(HCO_3). Au mirrors treated with (**1**-H)(HCO_3) via room temperature methanolic deposition (top), heated methanolic deposition at 55 °C (middle), and *in vacuo* deposition at 120 °C (bottom). For all of these systems, a N 1s signal between 399.9 eV and 401 eV in agreement with an NHC bound to gold was observed,[9-12] and an additional signal at >401 eV was also observed.[12-14]

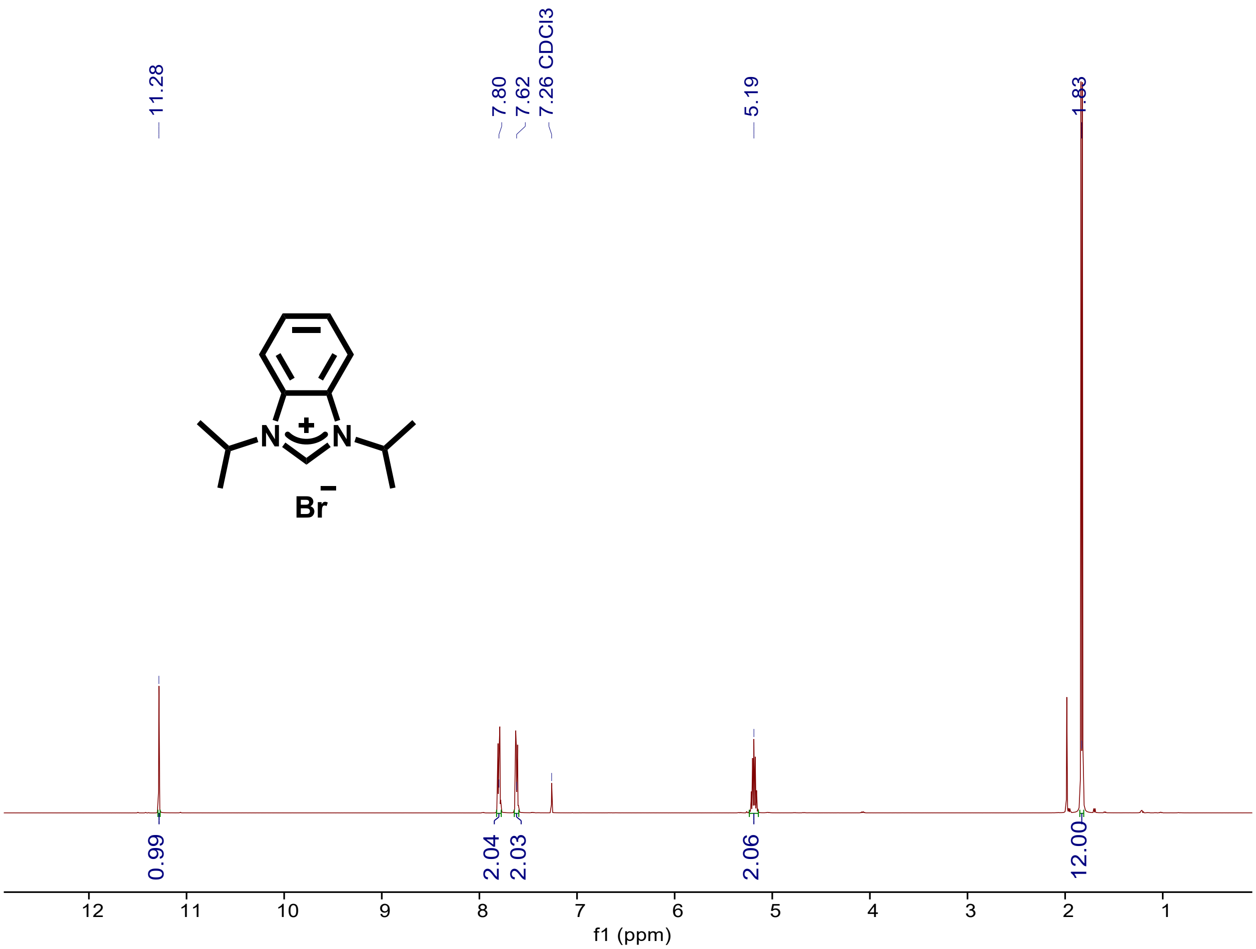

Figure S18. ^{1}H NMR spectrum of (**1**-H)(Br) in $CDCl_3$.

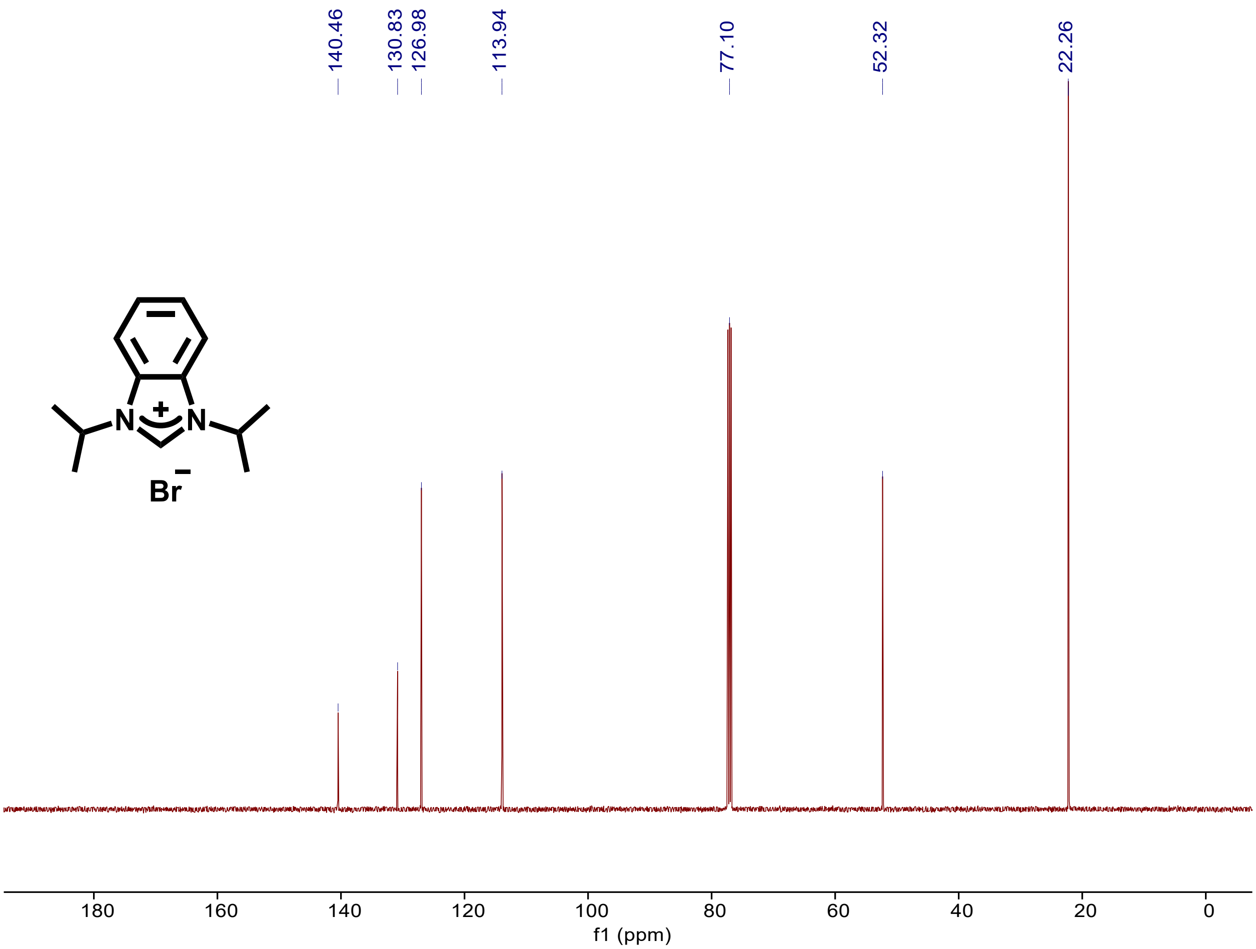

Figure S19. $^{13}C\{^{1}H\}$ NMR spectrum of (**1**-H)(Br) in $CDCl_3$.

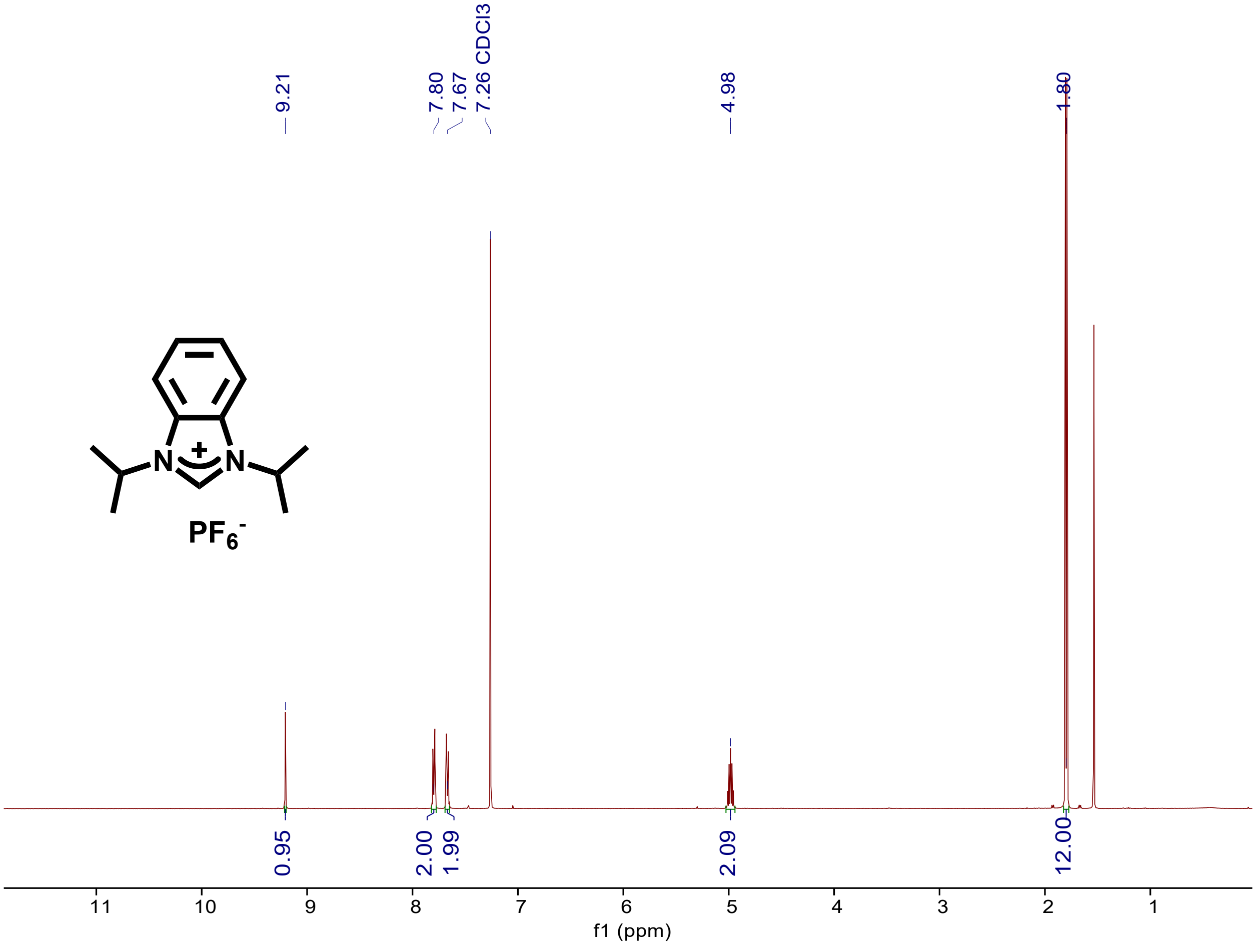

Figure S20. ^{1}H NMR spectrum of (**1**-H)(PF_6) in $CDCl_3$.

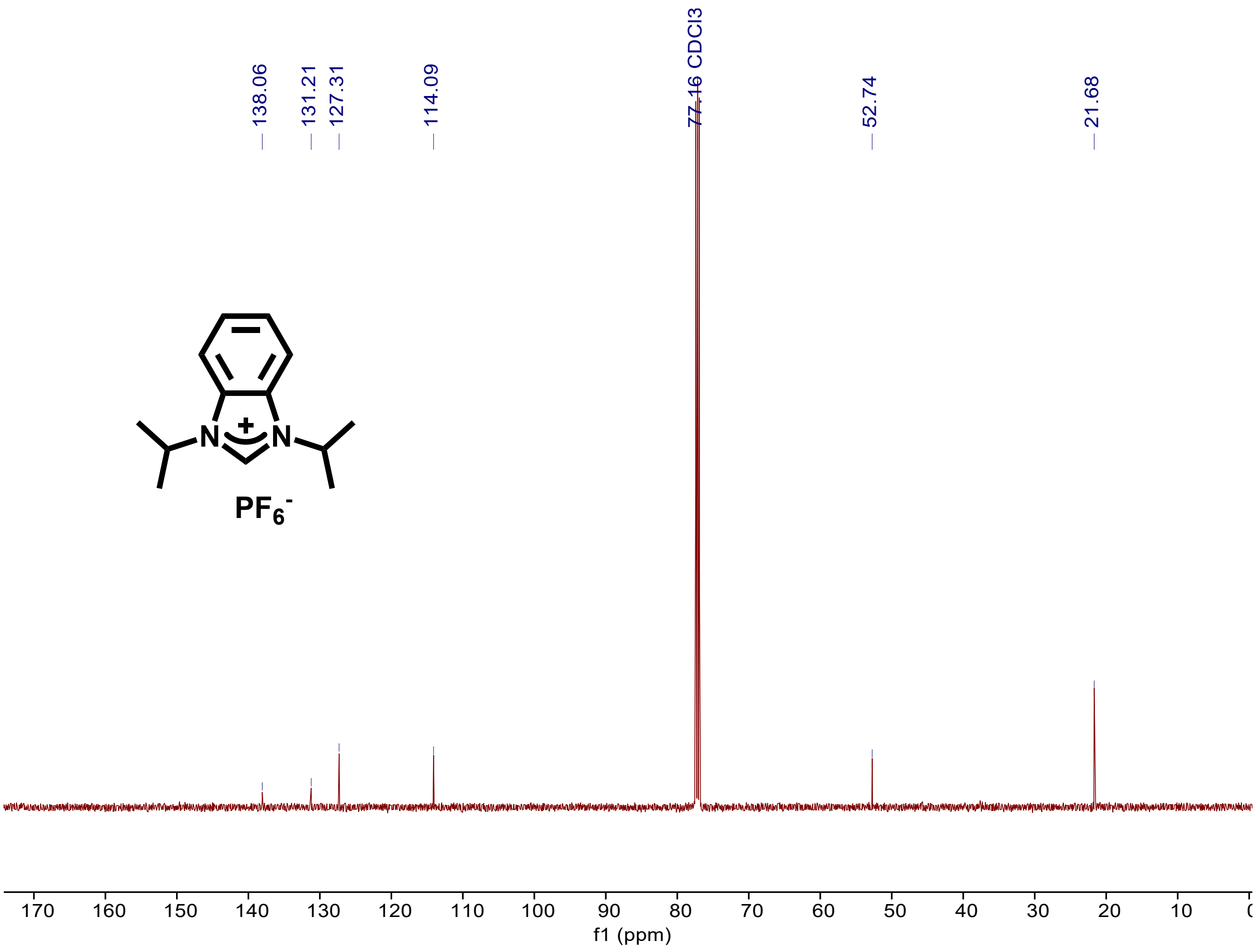

Figure S21. $^{13}C\{^{1}H\}$ NMR spectrum of (**1**-H)(PF_6) in $CDCl_3$.

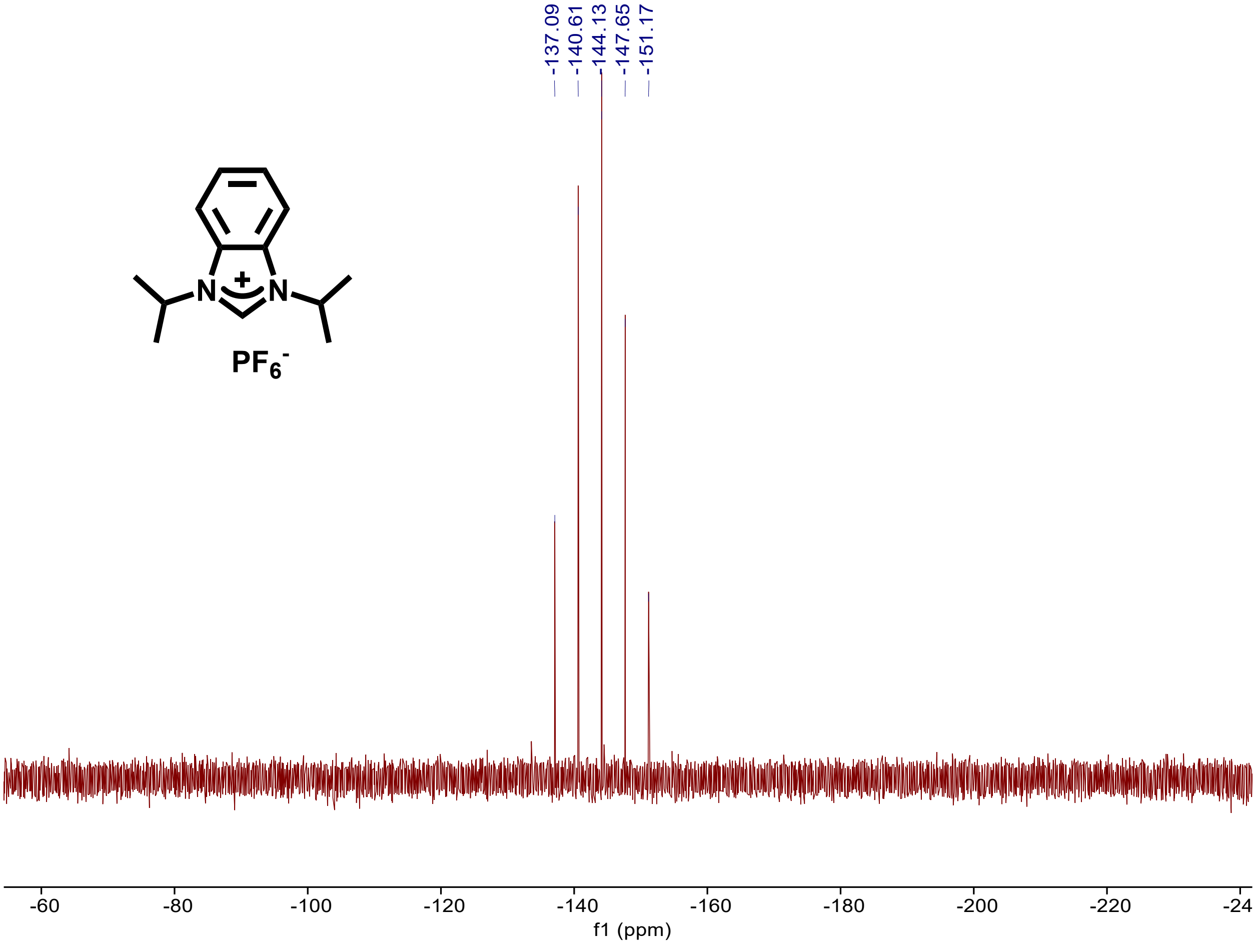

Figure S22. $^{31}P\{^{1}H\}$ NMR spectrum of (**1**-H)(PF_6).

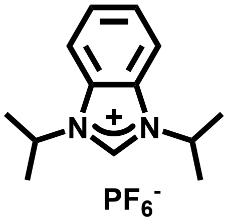

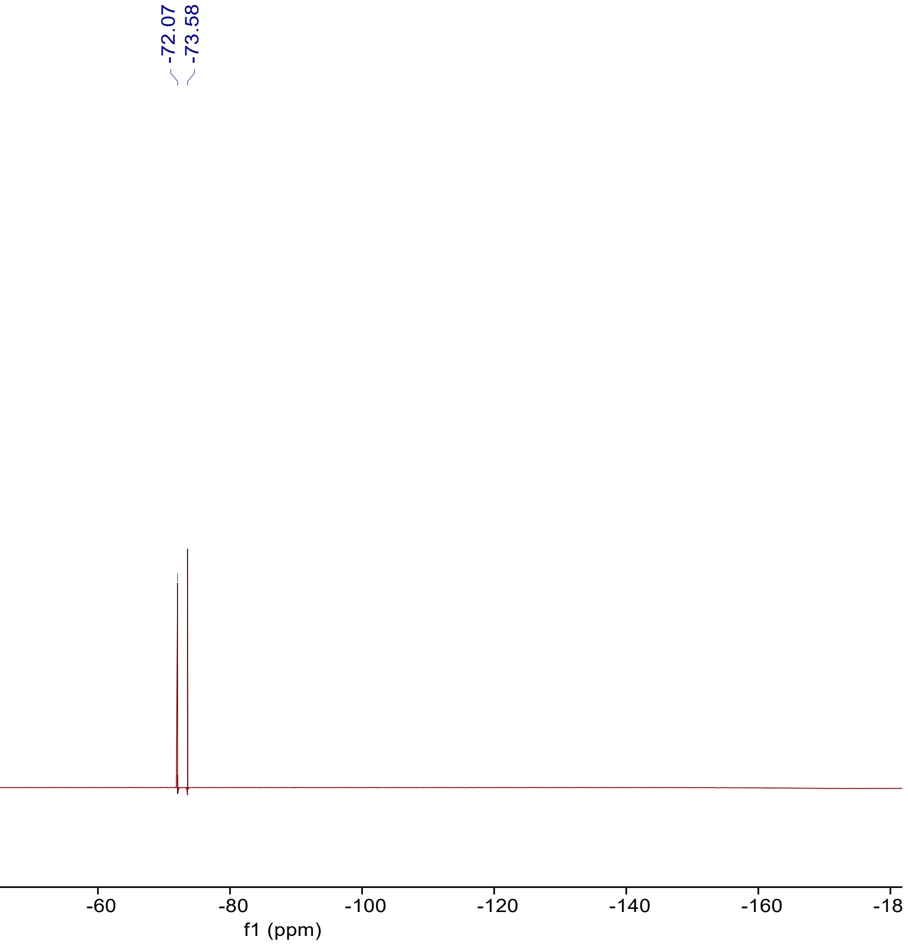

Figure S23. $^{19}F\{^{1}H\}$ NMR spectrum of (**1**-H)(PF_6).

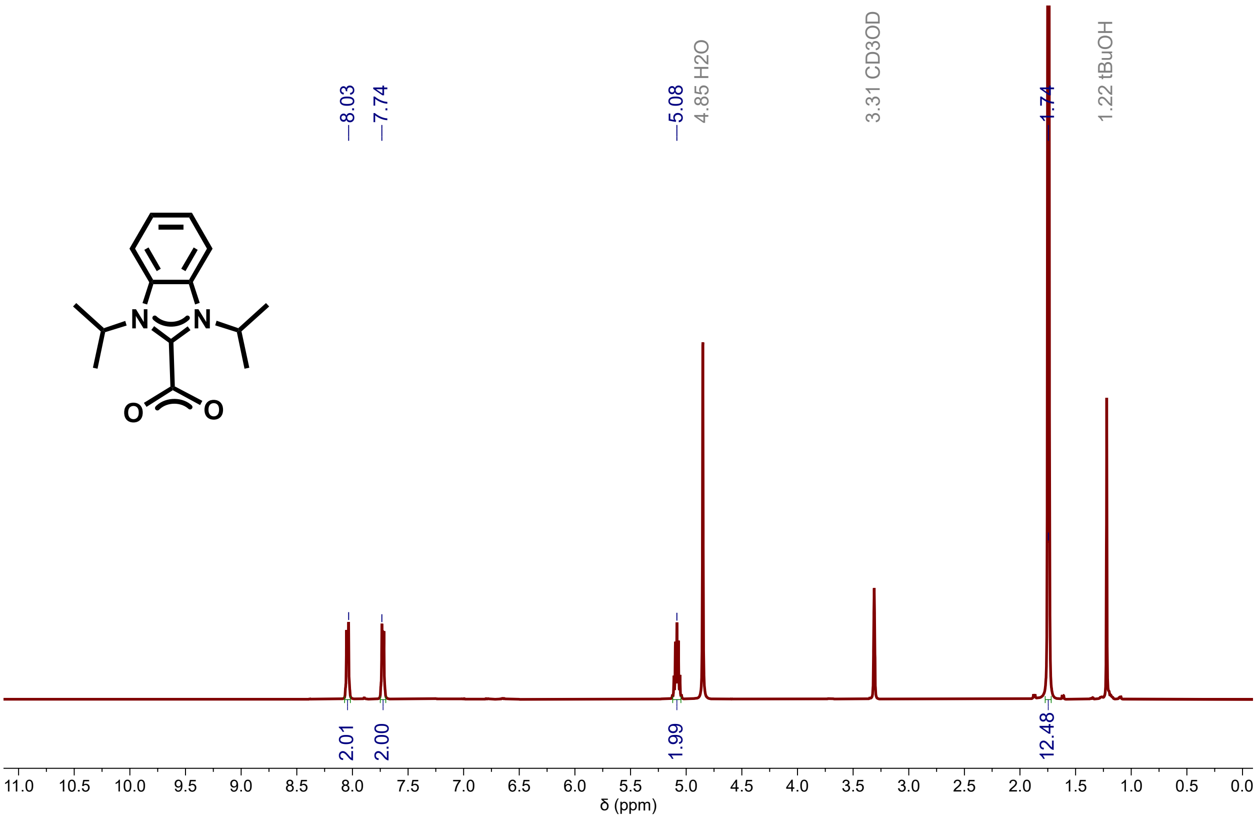

Figure S24. ^{1}H NMR spectrum of **1**-CO_2 in CD_3OD.

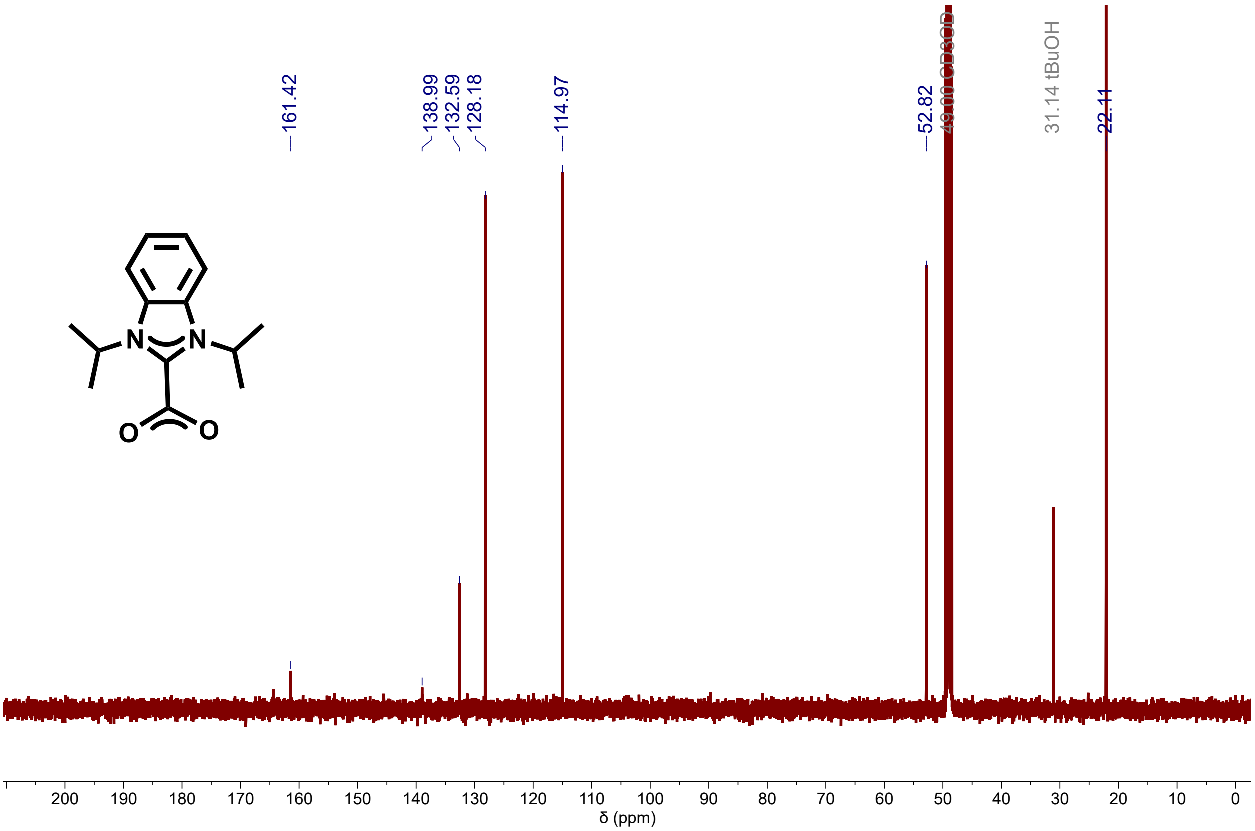

Figure S25. $^{13}C\{^{1}H\}$ NMR spectrum of **1**-CO_2 in CD_3OD.

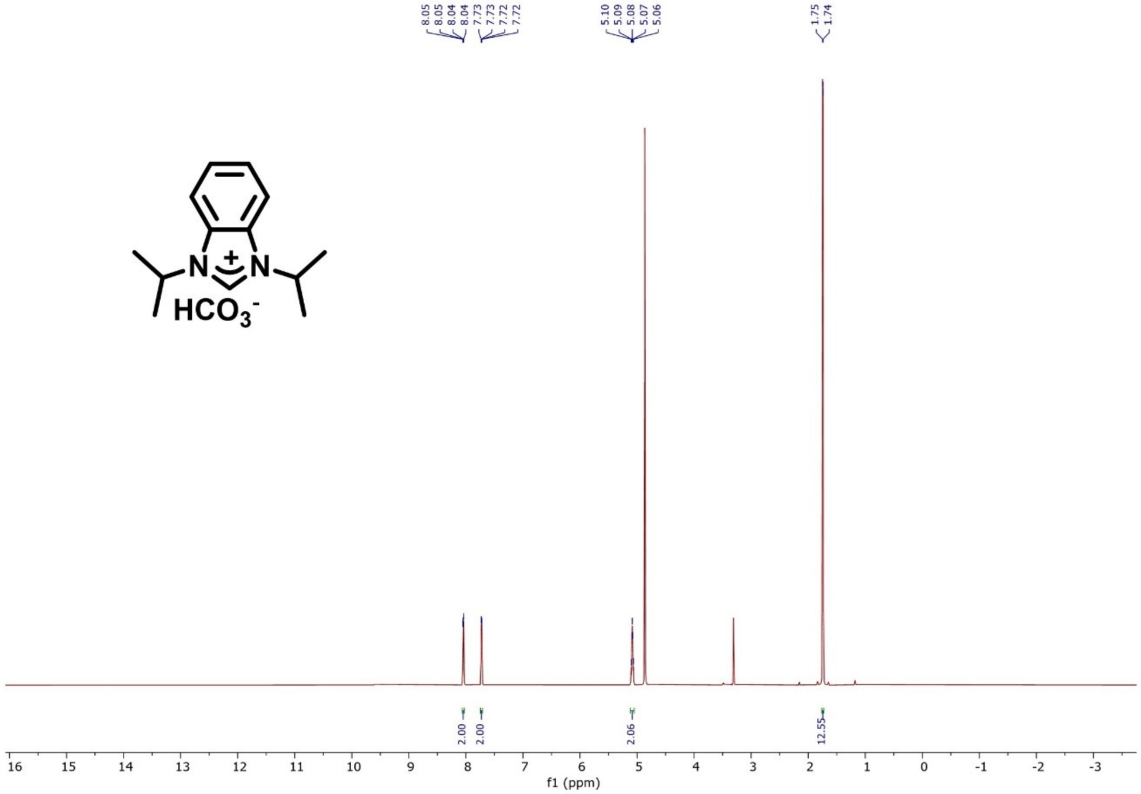

Figure S26. ^{1}H NMR spectrum of (**1**-H)(HCO_3).

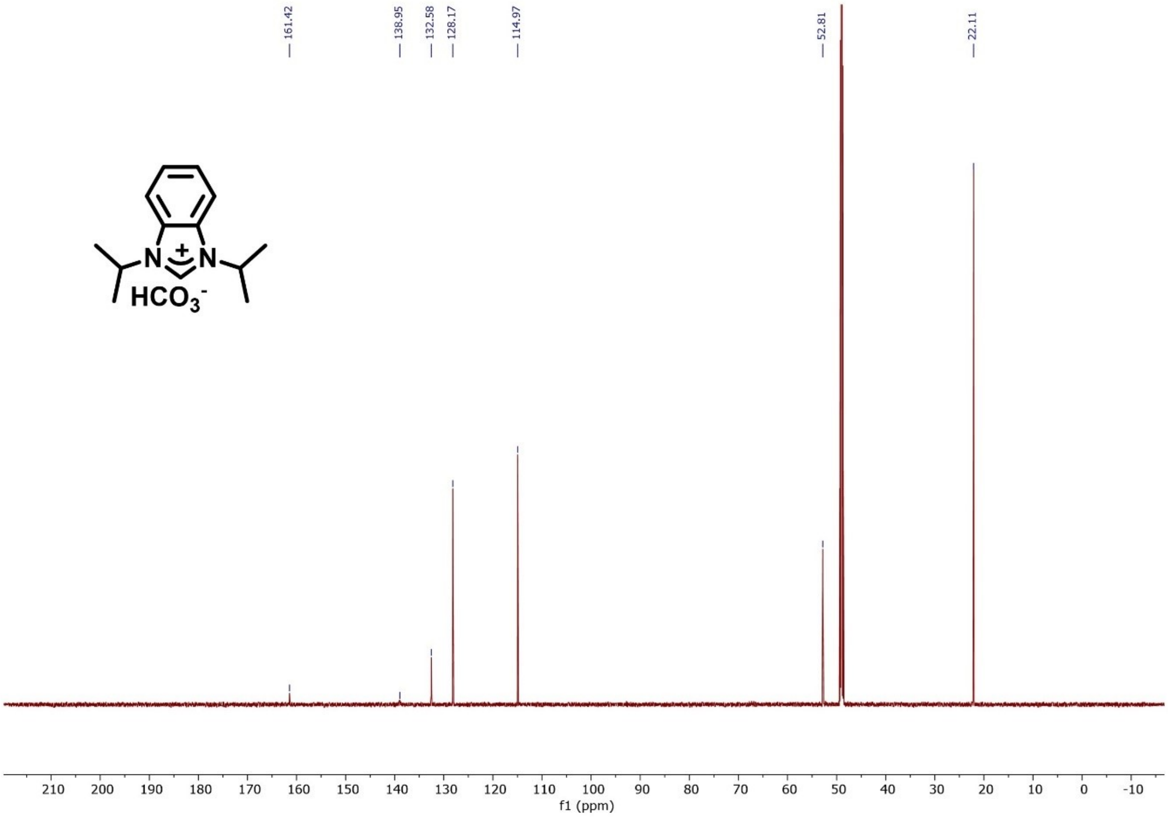

Figure S27. $^{13}C\{^1H\}$ NMR spectrum of (**1**-H)(HCO_3).

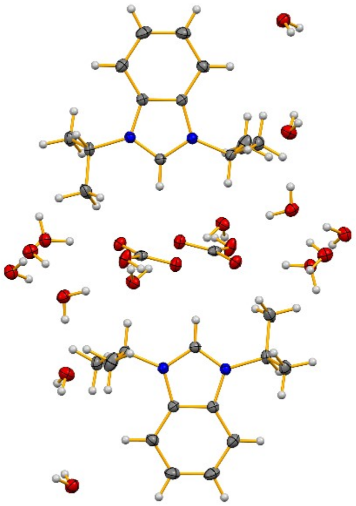

Figure S28. The Crystal Structure of (1-H)(HCO_3). (**1**-H)(HCO_3) was crystallized in the NMR tube during solvent evaporation under ambient condition with $CDCl_3$ as a solvent. Clear colorless block crystals were obtained and selected for SCXRD analysis. There are seven water molecules in the asymmetric unit indicating the molecule is highly hygroscopic.

Compound	**NHC-HCO3**
Empirical formula	$C_{14}H_{34}N_2O_{10}$
Formula weight	390.43
Temperature/K	100.00
Crystal system	triclinic
Space group	*P–1*
a/Å<br>b/Å<br>c/Å	8.0865(4)<br>11.5014(5)<br>12.6813(7)
α/°<br>β/°<br>γ/°	63.784(2)<br>86.101(2)<br>85.115(2)
Volume/$Å^3$	1053.66(9)
Z	2
$\rho_{calc}g/cm^3$	1.231
μ/mm^{-1}	0.886
F(000)	424.0
Crystal size/mm^3	0.534 × 0.2 × 0.026
Radiation	CuKα (λ = 1.54178)
2Θ range for data collection/°	7.776 to 136.624
Index ranges	-8 ≤ h ≤ 9<br>-13 ≤ k ≤ 13<br>-15 ≤ l ≤ 15
Reflections collected	16270
Independent reflections	3838<br>[R_{int} = 0.0355,<br>R_{sigma} = 0.0267]
Data/restraints/parameters	3838/0/293
Goodness-of-fit on F^2	1.130
Final R indexes [I>=2σ (I)]	R_1 = 0.0380,<br>wR_2 = 0.1001
Final R indexes [all data]	R_1 = 0.0405,<br>wR_2 = 0.1018
Largest diff. peak/hole / e $Å^{-3}$	0.21/-0.25

Table S1. Crystallographic data of (**1**-H)(HCO_3).

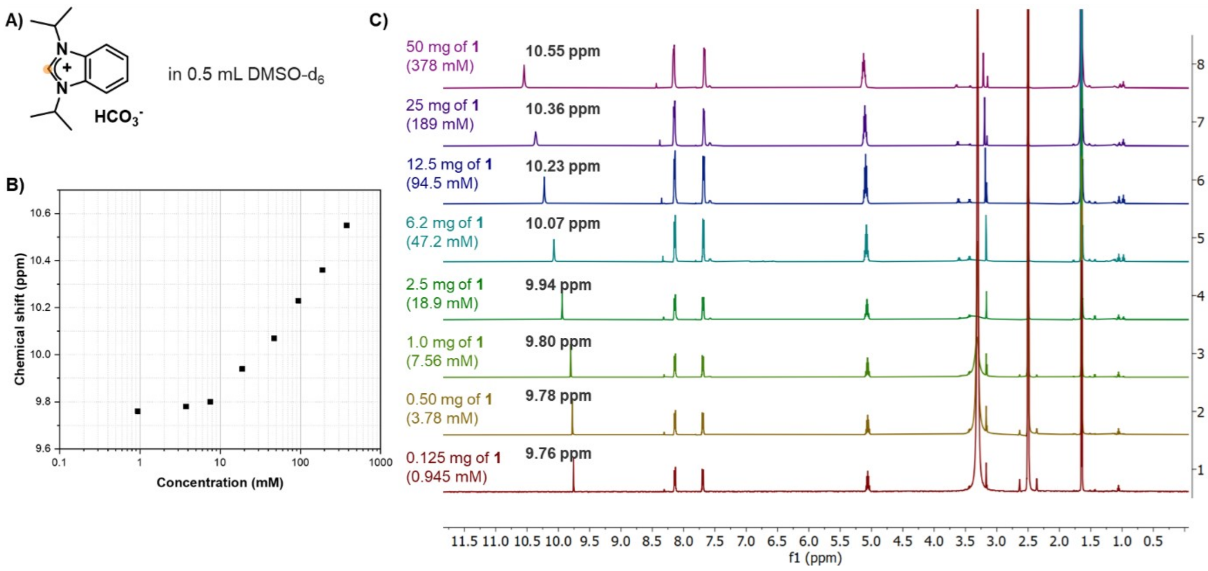

Figure S29. NMR of (1-H)(HCO_3) as a Function of Concentration. A) The structure of (**1**-H)(HCO_3) with the highlighting the 2-position with a yellow dot (carbene carbon)). B) Chemical shift of the proton at 2-position with respect to the concentration of (**1**-H)(HCO_3) in solution. C) The ^{1}H NMR spectra of (**1**-H)(HCO_3) at concentrations ranging from 0.945 mM to 378 mM.

References

(1) Greczynski, G.; Hultman, L. Compromising Science by Ignorant Instrument Calibration—Need to Revisit Half a Century of Published XPS Data. *Angewandte Chemie International Edition* **2020**, *59* (13), 5002-5006, DOI: https://doi.org/10.1002/anie.201916000.

(2) Greczynski, G.; Hultman, L. C 1s Peak of Adventitious Carbon Aligns to the Vacuum Level: Dire Consequences for Material's Bonding Assignment by Photoelectron Spectroscopy. *ChemPhysChem* **2017**, *18* (12), 1507-1512, DOI: https://doi.org/10.1002/cphc.201700126.

(3) Barr, T. L.; Seal, S. Nature of the use of adventitious carbon as a binding energy standard. *J Vac Sci Technol A* **1995**, *13* (3), 1239-1246, DOI: 10.1116/1.579868.

(4) Swift, P. Adventitious carbon—the panacea for energy referencing? *Surface and Interface Analysis* **1982**, *4* (2), 47-51, DOI: https://doi.org/10.1002/sia.740040204.

(5) Guella, G.; Ascenzi, D.; Franceschi, P.; Tosi, P. The intriguing case of organic impurities contained in synthetic methanol: a mass spectrometry based investigation. *Rapid Communications in Mass Spectrometry* **2007**, *21* (20), 3337-3344, DOI: https://doi.org/10.1002/rcm.3222.

(6) Deshmukh, B. K.; Coetzee, J. F. Characterization of reactive impurities in methanol, ethanol, and 2-propanol by monitoring the activities of added ionic probes with ion selective electrodes. *Analytical Chemistry* **1984**, *56* (13), 2373-2378, DOI: 10.1021/ac00277a026.

(7) Coetzee, J. F.; Deshmukh, B. K. Determination of trace amounts of basic impurities in nonaqueous solvents with ion-selective electrodes. *Analytical Chemistry* **1983**, *55* (14), 2422-2424, DOI: 10.1021/ac00264a050.

(8) Krzykawska, A.; Wrobel, M.; Koziel, K.; Cyganik, P. N-Heterocyclic Carbenes for the Self-Assembly of Thin and Highly Insulating Monolayers with High Quality and Stability. *ACS Nano* **2020**, *14* (5), 6043-6057, DOI: 10.1021/acsnano.0c01733.

(9) Inayeh, A.; Groome, R. R. K.; Singh, I.; Veinot, A. J.; de Lima, F. C.; Miwa, R. H.; Crudden, C. M.; McLean, A. B. Self-assembly of N-heterocyclic carbenes on Au(111). *Nat Commun* **2021**, *12* (1), 4034, DOI: 10.1038/s41467-021-23940-0.

(10) Bakker, A.; Freitag, M.; Kolodzeiski, E.; Bellotti, P.; Timmer, A.; Ren, J.; Schulze Lammers, B.; Moock, D.; Roesky, H. W.; Monig, H.; Amirjalayer, S.; Fuchs, H.; Glorius, F. An Electron-Rich Cyclic (Alkyl)(Amino)Carbene on Au(111), Ag(111), and Cu(111)

Surfaces. *Angew Chem Int Ed* **2020**, *59* (32), 13643-13646, DOI: 10.1002/anie.201915618.

(11) Nguyen, D. T.; Freitag, M.; Korsgen, M.; Lamping, S.; Ruhling, A.; Schafer, A. H.; Siekman, M. H.; Arlinghaus, H. F.; van der Wiel, W. G.; Glorius, F.; Ravoo, B. J. Versatile Micropatterns of N-Heterocyclic Carbenes on Gold Surfaces: Increased Thermal and Pattern Stability with Enhanced Conductivity. *Angew Chem Int Ed* **2018**, *57* (35), 11465-11469, DOI: 10.1002/anie.201807197.

(12) Gutheil, C.; Roß, G.; Amirjalayer, S.; Mo, B.; Schäfer, A. H.; Doltsinis, N. L.; Braunschweig, B.; Glorius, F. Tailored Monolayers of N-Heterocyclic Carbenes by Kinetic Control. *ACS Nano* **2024**, *18* (4), 3043-3052, DOI: 10.1021/acsnano.3c08045.

(13) Ruhling, A.; Schaepe, K.; Rakers, L.; Vonhoren, B.; Tegeder, P.; Ravoo, B. J.; Glorius, F. Modular Bidentate Hybrid NHC-Thioether Ligands for the Stabilization of Palladium Nanoparticles in Various Solvents. *Angew Chem Int Ed* **2016**, *55* (19), 5856-5860, DOI: 10.1002/anie.201508933.

(14) Nguyen, D. T.; Freitag, M.; Gutheil, C.; Sotthewes, K.; Tyler, B. J.; Bockmann, M.; Das, M.; Schluter, F.; Doltsinis, N. L.; Arlinghaus, H. F.; Ravoo, B. J.; Glorius, F. An Arylazopyrazole-Based N-Heterocyclic Carbene as a Photoswitch on Gold Surfaces: Light-Switchable Wettability, Work Function, and Conductance. *Angew Chem Int Ed* **2020**, *59* (32), 13651-13656, DOI: 10.1002/anie.202003523.
